# YAP promotes the proliferation of neuroblastoma cells through decreasing the nuclear location of p27^Kip1^ mediated by Akt

**DOI:** 10.1111/cpr.12734

**Published:** 2019-12-20

**Authors:** Xiya Shen, Xingxing Xu, Changnan Xie, Huitao Liu, Danlu Yang, Jingjing Zhang, Qian Wu, Wenjin Feng, Ling Wang, Leilei Du, Lina Xuan, Chaobo Meng, Haitao Zhang, Wei Wang, Ying Wang, Tian Xie, Zhihui Huang

**Affiliations:** ^1^ School of Basic Medical Sciences Wenzhou Medical University Wenzhou China; ^2^ Key Laboratory of Elemene Anti‐cancer Medicine of Zhejiang Province and Holistic Integrative Pharmacy Institutes Hangzhou Normal University Hangzhou China; ^3^ Engineering Laboratory of Development and Application of Traditional Chinese Medicine from Zhejiang Province and Holistic Integrative Pharmacy Institutes Hangzhou Normal University Hangzhou China; ^4^ Department of Spine Surgery The First Affiliated Hospital of Wenzhou Medical University Wenzhou China; ^5^ School of Mental Health Wenzhou Medical University Zhejiang China; ^6^ Zhejiang Sinogen Medical Equipment Co., Ltd. Wenzhou Zhejiang China; ^7^ Department of Neurobiology Key Laboratory of Medical Neurobiology (Ministry of Health of China) Collaborative Innovation Center for Brain Science Zhejiang University School of Medicine Hangzhou China; ^8^ MOE Key Laboratory of Biosystems Homeostasis and Protection and Innovation Center for Cell Signaling Network Life Sciences Institute Zhejiang University Hangzhou China; ^9^ Department of Transfusion Medicine Zhejiang Provincial People's Hospital of Hangzhou Medical College Hangzhou China

**Keywords:** nuclear location, p27^Kip1^, proliferation, SH‐SY5Y, YAP

## Abstract

**Objective:**

We aimed to investigate the roles and underlying mechanisms of YAP in the proliferation of neuroblastoma cells.

**Methods:**

The expression level of YAP was evaluated by Western blotting and immunocytochemistry. Cell viability, cell proliferation and growth were detected by CCK‐8, PH3 and Ki67 immunostaining, and the real‐time cell analyser system. The nuclear and cytoplasmic proteins of p27^Kip1^ were dissociated by the nuclear‐cytosol extraction kit and were detected by Western blotting and immunocytochemistry. mRNA levels of Akt, CDK5 and CRM1 were determined by qRT‐PCR.

**Results:**

YAP was enriched in SH‐SY5Y cells (a human neuroblastoma cell line). Knock‐down of YAP in SH‐SY5Y cells or SK‐N‐SH cell line (another human neuroblastoma cell line) significantly decreased cell viability, inhibited cell proliferation and growth. Mechanistically, knock‐down of YAP increased the nuclear location of p27^Kip1^, whereas serum‐induced YAP activation decreased the nuclear location of p27^Kip1^ and was required for cell proliferation. Meanwhile, overexpression of YAP in these serum‐starved SH‐SY5Y cells decreased the nuclear location of p27^Kip1^, promoted cell proliferation and overexpression of p27^Kip1^ in YAP‐activated cells inhibited cell proliferation. Furthermore, knock‐down of YAP reduced Akt mRNA and protein levels. Overexpression of Akt in YAP‐downregulated cells decreased the nuclear location of p27^Kip1^ and accelerated the proliferation of SH‐SY5Y cells.

**Conclusions:**

Our studies suggest that YAP promotes the proliferation of neuroblastoma cells through negatively controlling the nuclear location of p27^Kip1^ mediated by Akt.

## 1 INTRODUCTION

The Hippo signalling pathway is a critical regulator of stem cell self‐renewal, tissue regeneration and organ size.^1^, ^2^, ^3^, ^4^, ^5^ Dysfunction of this classical pathway will lead to several diseases such as tumours including lung cancer, liver cancer, breast cancer, colorectal cancer and gliomas.^6^, ^7^ As the principle effector of the Hippo pathway and a key transcriptional co‐factor, YAP plays important roles in organ size control through regulating cell differentiation and proliferation.^8^, ^9^, ^10^ Uncontrolled cell proliferation caused by excessive YAP activation will give rise to brain tumours that are usually fatal, such as neuroblastoma and medulloblastoma.^11^, ^12^, ^13^

Neuroblastoma (NB) is one of the most common extracranial solid tumours in children.^14^ So far, the role of YAP in neuroblastoma has rarely been studied.^13^ In recent decades, YAP is found to participate in the proliferation of neuroblastoma. Some studies have indirectly suggested that YAP and TAZ are activated in neuroblastoma and may be closely related to neuroblastoma invasion and metastasis.^15^, ^16^ For example, in recurrent neuroblastoma, a mutation in PTPN14 as a negative regulator of YAP is detected, thus, YAP activity may be increased in recurrent neuroblastoma.^15^ TAZ can promote the transformation of epithelial cells into mesenchymal cells and promote neuroblastoma invasion and metastasis.^16^ As a co‐transcription factor of TAZ, YAP may also have the similar effects on the invasion and metastasis of neuroblastoma. Moreover, YAP is highly expressed in neuroblastoma and the expression level is correlated with advanced tumour staging. Downregulation of YAP significantly impairs neuroblastoma proliferation, tumorigenesis and invasion in vitro.^13^ In addition, injection of the YAP inhibitor, peptide 17, dramatically prevents the subcutaneous tumour growth of neuroblastoma.^13^ Although these evidences indicate that YAP promotes the tumorigenesis of neuroblastoma, the detailed mechanism about how YAP modulates the proliferation of neuroblastoma cells remains unclear.

Cell proliferation is closely related to the cell cycle. Cyclin‐dependent kinases inhibitors (CDKIs), such as p27^Kip1^ and p21^Cip1^, by binding to CDK, inhibit the kinase activity of most CDKs, thereby inhibit cell proliferation and exert anti‐tumour activity.^17^ There are evidences showing that p27^kip1^ is related to neuroblastoma as well as p16, p21 and p53.^18^, ^19^, ^20^, ^21^ Decreased transcript levels of p27^Kip1^ increases human susceptibility to neuroblastoma,^22^ positive expression of p27^Kip1^ increases survival in patients with neuroblastoma^18^ and accumulation of p27^Kip1^ inhibits the growth of human neuroblastoma cells.^23^ Several studies have shown that p27^Kip1^ can be an important regulatory target of YAP, affecting cell proliferation; however, the conclusions seem to be contradictory to each other. It is reported that exogenous YAP could induce cell proliferation, enhance cyclin D1 expression and reduce p27^kip1^/p21^cip1^ levels in contact‐inhibited HCEC monolayers and post‐confluent B4G12 cells.^24^ This means that low p27^kip1^ level is conducive to cell proliferation, which is the widely accepted view.^25^, ^26^, ^27^ However, there is also evidence showing that low p27^kip1^ level can cause defect in cell cycle progression, and the expression of p27^kip1^ can also be regulated by YAP.^28^

The nuclear and cytoplasmic location of p27^kip1^ might be the critical reason that affects cell proliferation, rather than the total p27^kip1^ expression.^29^, ^30^, ^31^ As an inhibitor of DNA duplication and cell division, p27^Kip1^ protein locates in the cytoplasm as well as in the nucleus and exerts its anti‐proliferative action inside the nucleus. Accumulation of nuclear p27^Kip1^ prevents cell proliferation.^29^, ^32^ However, it remains unclear whether YAP regulates the proliferation of neuroblastoma through controlling the nuclear distribution of p27^Kip1^.

In this study, we found YAP was enriched in SH‐SY5Y cells, a cell line of neuroblastoma. Knock‐down of YAP in SH‐SY5Y cells slowed down cell proliferation, reduced Akt mRNA and protein levels and increased the nuclear location of p27^Kip1^. Overexpression of Akt in YAP‐inactivated cells decreased the nuclear location of p27^Kip1^ and increased cell proliferation. Our results suggest that YAP regulates the proliferation of neuroblastoma cells through decreasing the nuclear distribution of p27^Kip1^ via Akt. Therefore, our findings suggest that nuclear p27^Kip1^ entrapment by targeting YAP‐Akt signalling may be a potential therapeutic strategy for neuroblastoma.

## 2 MATERIALS AND METHODS

### 2.1 cDNA constructs

YAP‐shRNA and control‐shRNA plasmids were kindly provided by Prof. Bin Zhao (Zhejiang University), as described before.^33^ We used human pEGFP, pEGFP‐hYAP1 and pEGFP‐p27^Kip1^ cDNA vectors for expression and rescue experiments. The pEGFP‐p27^Kip1^ plasmid is gifted by Prof. Chuanshu Huang (New York University School of Medicine). pEGFP‐hYAP1 (http://www.addgene.org/17843) and pEGFP‐Akt1 (http://www.addgene.org/86637/) were purchased from Addgene. The sequence of YAPi‐1 siRNA was 5′‐CCGGGATGACTCAGGAATT‐3′; the sequence of YAPi‐2 siRNA was 5′‐GGCAATACGGAATATCAAT‐3′; the sequence of YAPi‐3 siRNA was 5′‐GGAGAGGCTGCGATTGAAA‐3′; and these sequences were inserted into pSurper vectors under the control of H1 promoter. All these expression constructs were verified by sequencing and tested for normal expression by Western blotting, as shown in Figures S1 and S2.

### 2.2 Antibodies

**Table:** 

Antibody	Company	Catalog	Dilution
YAP	Abcam	ab205270	WB 1:1000
YAP	Sigma‐Aldrich	WH0010413M1	WB 1:1000; staining 1:200
GFAP	Millipore	MAB360	Staining 1:200
p‐YAP	Cell Signaling Technology	13008	WB 1:1000
PH3	Abcam	ab14955	Staining 1:2500
PH3	Millipore	06‐570	Staining 1:200
Ki67	Thermo scientific	RM‐9106	Staining 1:200
Ki67	Millipore	AB9260	Staining 1:200
p27^Kip1^	Cell Signaling Technology	3698	WB 1:1000
p27^Kip1^	BD Biosciences	610242	Staining 1:200
p‐Akt‐Thr308	Cell Signaling Technology	13038	WB 1:1000
p‐p27^kip1^‐Ser10	Abcam	Ab62364	WB 1:1000
Lamin B1	Abcam	ab16048	WB 1:1000
β‐actin	Sigma‐Aldrich	A5316	WB 1:10 000
GAPDH	Cell Signaling	2118	WB 1:5000
Anti‐mouse IgG‐HRP	Pierce	31460	1:10 000
Anti‐rabbit IgG‐HRP	Pierce	31420	1:10 000
Anti‐rabbit Alexa Fluor488	Invitrogen	A21206	1:1000
Anti‐mouse Alexa Fluor488	Invitrogen	A21202	1:1000
Anti‐rabbit Alexa Fluor546	Invitrogen	A10040	1:1000
Anti‐mouse Alexa Fluor546	Invitrogen	A10036	1:1000

### 2.3 Cell culture and transfection

Primary astrocyte cultures were prepared from the cerebral cortex of P1‐P3 mice as described previously.^9^ In brief, cerebral neocortex was dissected, chopped, and then incubated with 0.125% trypsin (Gibco) at 37°C for 15‐20 minutes, and dissociated into a single‐cell suspension by mechanical disruption. The cells were seeded on poly‐L‐lysine (0.1 mg/mL; Sigma‐Aldrich)‐coated culture flasks and cultured with DMEM containing 10% foetal bovine serum (FBS, Gibco). After 6‐10 days, microglia and oligodendrocytes were removed by shaking at 250 rpm for 4‐6 hours. Astrocytes were subsequently detached and plated into poly‐D‐lysine‐coated dishes or coverslips. The purity of GFAP‐positive cells in our culture system was more than 94%.

SH‐SY5Y, A172, U87 and DBTRG cell lines were gifted by Prof. Maojin Yao (Sun Yat‐Sen University) and were grown in a humidified atmosphere of 5% CO2 at 37°C. SK‐N‐SH were purchased from COBIOER BIOSCIENCES CO., LTD. Human neuroblastoma cells (SH‐SY5Y) and human glioma cells (A172, U87), were grown in DMEM, supplemented with 10% FBS (Gibco) and 1% penicillin/streptomycin (Gibco). The human glioma cell line, DBTRG cells, was grown in RPMI‐1640 medium (Gibco), supplemented with 10% FBS (Gibco), 1% penicillin/streptomycin (Gibco) and 2 mmol/L l‐glutamine (Invitrogen). Another Human neuroblastoma cells (SK‐N‐SH) were grown in MEM, supplemented with 10% FBS, 1% penicillin/streptomycin, 1% GlutaMax, 1% NEAA and 1% sodium pyruvate. Appropriate plasmids (2 μg per 35‐mm dish) were transfected into the cells using Lipofectamine™ 3000 Transfection Reagent (L3000‐015; Invitrogen) according to the manufacturer's protocol. 48‐72 hours after transfection, cells were used for experiments.

### 2.4 Western blotting

Western blotting was carried out as described previously.^9^ Briefly, cultured cells were lysed by ice‐cold RIPA Buffer (P0013B; Beyotime) and incubated at 4°C for 30 minutes. Following centrifugation at 12 000 × *g* for 10 minutes, proteins were extracted with 5× loading buffer and boiled at 100°C for 8‐10 minutes. The protein samples then were separated using 10% sodium dodecyl sulphate‐polyacrylamide gel electrophoresis (SDS‐PAGE) and were transferred onto nitrocellulose membranes (Life Sciences). After blocking in TBST containing 5% skim milk for 1 hour, the immunoblots were incubated with different primary antibodies as shown in above tables at 4°C overnight. Subsequently, the membranes were washed three times in TBST, and incubated with the horseradish peroxidase (HRP)‐conjugated secondary antibodies for 1 hour. After washing in TBST for another three times, the protein signals were detected using the ECL detection kit (Bio‐Rad). Blots were analysed using Quantity One software (Bio‐Rad).

### 2.5 Immunocytochemistry

The protocols used for immunofluorescence staining and quantitative analysis were described previously.^9^ Briefly, cultured cells were rinsed once with PBS, fixed in 4% paraformaldehyde for 20 minutes. Then, they were blocked and permeabilized with 0.1% Triton X‐100 in PBS containing 5% bovine serum albumin (BSA) at room temperature for 1 hour. Subsequently, cells were incubated with primary antibodies as shown above tables at 4°C overnight, washed three times in PBS and then with secondary antibodies at room temperature for 1 hour. After washing in PBS for another three times, cells were mounted. Images were acquired by using a fluorescence microscopy (NIKON). The density of fluorescence was measured by Image J software.

### 2.6 Cell counting Kit‐8 (CCK‐8) assay

Cell viability was measured by using CCK‐8 cell counting kit (A311‐01/02; Vazyme Biotech). In brief, the transfected SH‐SY5Y cells were seeded into 96‐well plates at a density of 2000 cells/well and cultured for 24‐48 hours. Subsequently, 10 μL CCK‐8 solution was added to each well and incubated at 37°C for 2 hours. The optical density at 450 nm, which was indicative of a positive correlation with cell viability, was measured using a microplate reader (Varioskan Flash; Thermo Scientific).

### 2.7 Growth curve

The growth curves for SH‐SY5Y cells transfected with control‐shRNA or YAP‐shRNA were generated by using the real‐time cell analyser system (IncuCyte S3). The atmosphere was maintained at 37°C, 95% O_2_ and 5% CO_2_ during recordings. Briefly, about 2‐4 × 10^5^ viable cells were seeded per well of a six‐well plate and recorded for 48 hours. Data were reported as confluence and were defined as the percentage of the cell density at different time points over the cell density at 48 hours, which was auto‐calculated by the offline software of IncuCyte S3.

### 2.8 RNA extraction and quantitative real‐time PCR (qRT‐PCR)

To determine the mRNA expression levels of genes, total RNA was extracted from cells using TRIzol™ reagent (15596026; Ambion) according to the protocol provided by the manufacturer. A total of 2 μg RNA was reversely transcribed into cDNA with a SuperScript™ One‐Step Reverse Transcription Kit (10928‐034; Invitrogen). The mRNA levels were quantified using the iTaq™ Universal SYBR® Green Supermix (172‐5122; Bio‐Rad) on the Real‐Time PCR detection System (Applied Biosystems). *β*‐actin was chosen as the endogenous control. The relative levels of mRNA expression were represented as Δ*C*t = *C*t gene‐*C*t reference, and the fold change of gene expression was calculated using the 2^−ΔΔ^ *^C^* ^t^ method. The primers used in this study were synthesized by Sangon Biotech and presented as follows^28^, ^34^, ^35^: *Akt*, 5′‐ATGGCACCTTCATTGGCTAC‐3′ and 5′‐CCCAGCAGCTTCAGGTACTC‐3′; *CDK5*, 5′‐CGCCGCGATGCAGAAATACGAGAA‐3′ and 5′‐TGGCCCCAAAGAGGACATC‐3′; *CRM1*, 5′‐CTCGTCAGCTGCTTGATTTC‐3′ and 5′‐CTCTTGTCCAAGCATCAGGA‐3′; *β*‐actin, 5′‐ATAGCACAGCCTGGATAGCAACGTAC‐3′ and 5′‐CACCTTCTACAATGAGCTGCGTGTG‐3′.

### 2.9 Cytosol‐nuclei fractionation

We used the nuclear‐cytosol extraction kit to dissociate the cytoplasmic and nuclear proteins (#P1200; Applygen) according to the manufacturer's instructions. Fractions were analysed by SDS‐PAGE and Western blot with specific antibodies.

### 2.10 Statistical analysis

All data values were presented as mean ± SEM derived from at least three independent experiments. GraphPad Prism software was used for statistical analysis. For comparison between two groups, we used unpaired t test; for comparison between three groups, we applied one‐way ANOVA with the Bonferroni post hoc multiple comparison test; for the analysis of the growth curve, two‐way ANOVA was utilized. A *P* value of <.05 was considered to be statistically significant.

## 3 RESULTS

### 3.1 YAP was enriched in the neuroblastoma cell line

**Figure 1:**
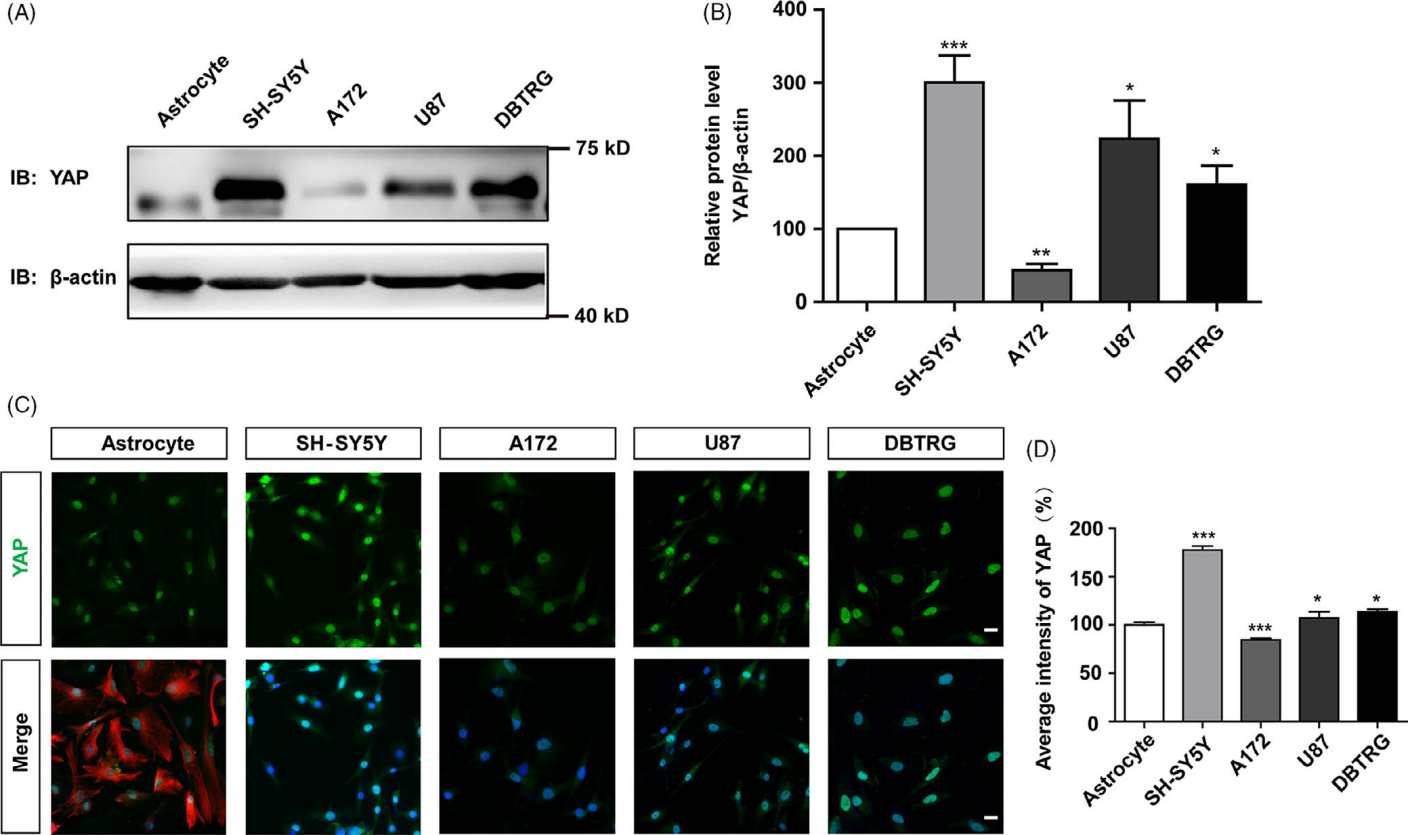
YAP was enriched in the neuroblastoma cell lines. A, Representative images of the expression of YAP detected by Western blot in cultured astrocytes and SH‐SY5Y, A172, U87, DBTRG glioma cell lines. B, Quantification of YAP expression in A (n = 3). C, Immunostaining of YAP (green) and GFAP (red) in cultured astrocytes and immunostaining of YAP (green) in SH‐SY5Y, A172, U87, DBTRG glioma cell lines. D, Quantification of the average intensity of YAP in C (n = 15). Scale bars, 20 μm. Data were mean ± SEM. *P < .05, **P < .01, ***P < .001

To examine the roles of YAP in neuroblastoma cells, we firstly detected the expression level of YAP proteins in SH‐SY5Y cells and control cells, such as astrocytes and three human glioma cell lines, A172 cells, U87 cells and DBTRG cells. Our previous studies have shown that YAP was highly expressed in cultured astrocytes.^8^ As shown in Figure 1A,B, Western blot results showed that YAP was highly expressed in SH‐SY5Y cells and was significantly higher than the primary cultured astrocytes and other glioma cell lines. Further immunostaining results showed that YAP was mainly expressed in the nucleus of SH‐SY5Y cells, astrocytes and other glioma cell lines (Figure 1C,D). These results suggest that YAP is enriched in the neuroblastoma cell line, which may be involved in the proliferation of neuroblastoma cells.

### 3.2 Knock‐down of YAP reduced the proliferation of SH‐SY5Y cells

**Figure 2:**
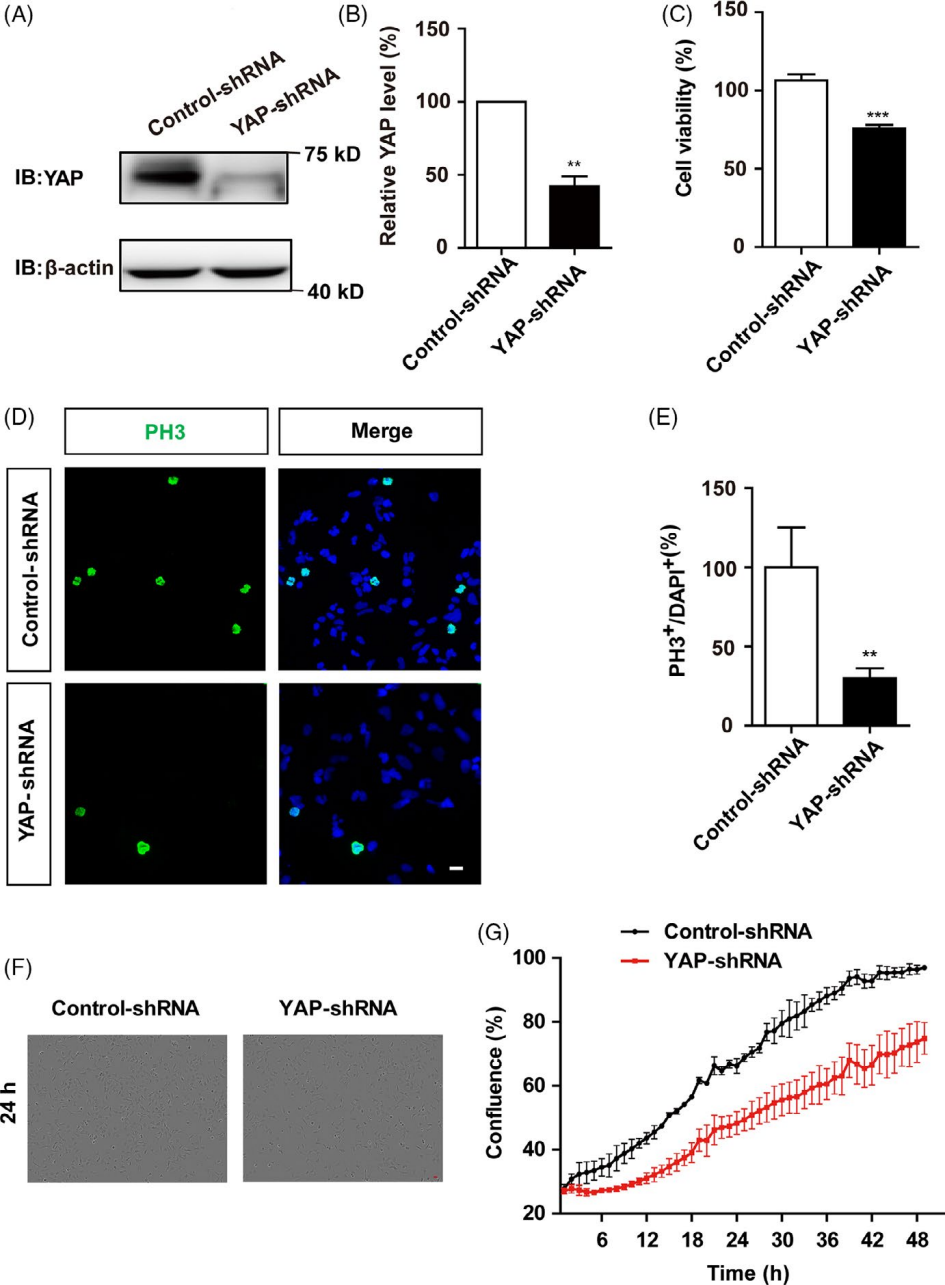
Knock‐down of YAP reduced the proliferation of SH‐SY5Y cells. A, Western blot analysis of the knock‐down efficiency of YAP. SH‐SY5Y cells were transfected with vectors encoding control‐shRNA or YAP‐shRNA for 48‐72 h. B, Quantification of YAP expression as shown in A (n = 3). C, The effects of YAP knock‐down on SH‐SY5Y cell viability detected by CCK‐8 (n = 3). D, Immunostaining analysis of PH3 (green) in SH‐SY5Y cells transfected with control‐shRNA and YAP‐shRNA for 48 h. E, Quantitative analysis of the percentages of PH3^+^ cells over total SH‐SY5Y cells as shown in D (n = 15). F, Representative bright field images of cell density of SH‐SY5Y cells transfected with control‐shRNA or YAP‐shRNA by using the real‐time cell analyser system at the time point of 24 h. G, Quantitative analysis of SH‐SY5Y cells transfected with control‐shRNA or YAP‐shRNA by using the real‐time cell analyser system measured at different time points (n = 3). Scale bars, 20 μm. Data were mean ± SEM. **P < .01, ***P < .001

To examine whether YAP was required for the proliferation of SH‐SY5Y cells, SH‐SY5Y cells were transfected with YAP‐shRNA constructs to downregulate YAP expression. As shown in Figure 2A,B and Figure S1, the protein level of YAP was decreased significantly by YAP‐shRNA constructs, compared with control‐shRNA constructs. Interestingly, knock‐down of YAP significantly decreased SH‐SY5Y cell viability (Figure 2C) and dramatically reduced the percentage of PH3 (a marker of cell proliferation) positive cells (Figure 2D,E), suggesting that YAP knock‐down reduced the proliferation of SH‐SY5Y cells. To further confirm this phenotype, growth curves of SH‐SY5Y cells were measured by real‐time cellular analysis, which is a non‐invasive method that can record the real‐time growth state of live cells and perform quantitative analysis while imaging. As shown in Figure 2F,G, the growth curves showed that knock‐down of YAP significantly reduced SH‐SY5Y cells growth. To exclude the non‐specific targeting, three other YAP‐shRNAs were designed, and they all downregulated YAP efficiently (Figure S2A,B). Moreover, one of these YAP‐shRNAs, YAPi‐3, significantly decreased SH‐SY5Y cell viability (Figure S2C) and reduced the percentage of Ki67 (a marker of cell proliferation) positive cells (Figure S2D‐F). These results strongly suggest that YAP promotes the proliferation of SH‐SY5Y cells.

### 3.3 Knock‐down of YAP increased the nuclear location of p27^Kip1^ in SH‐SY5Y cells

**Figure 3:**
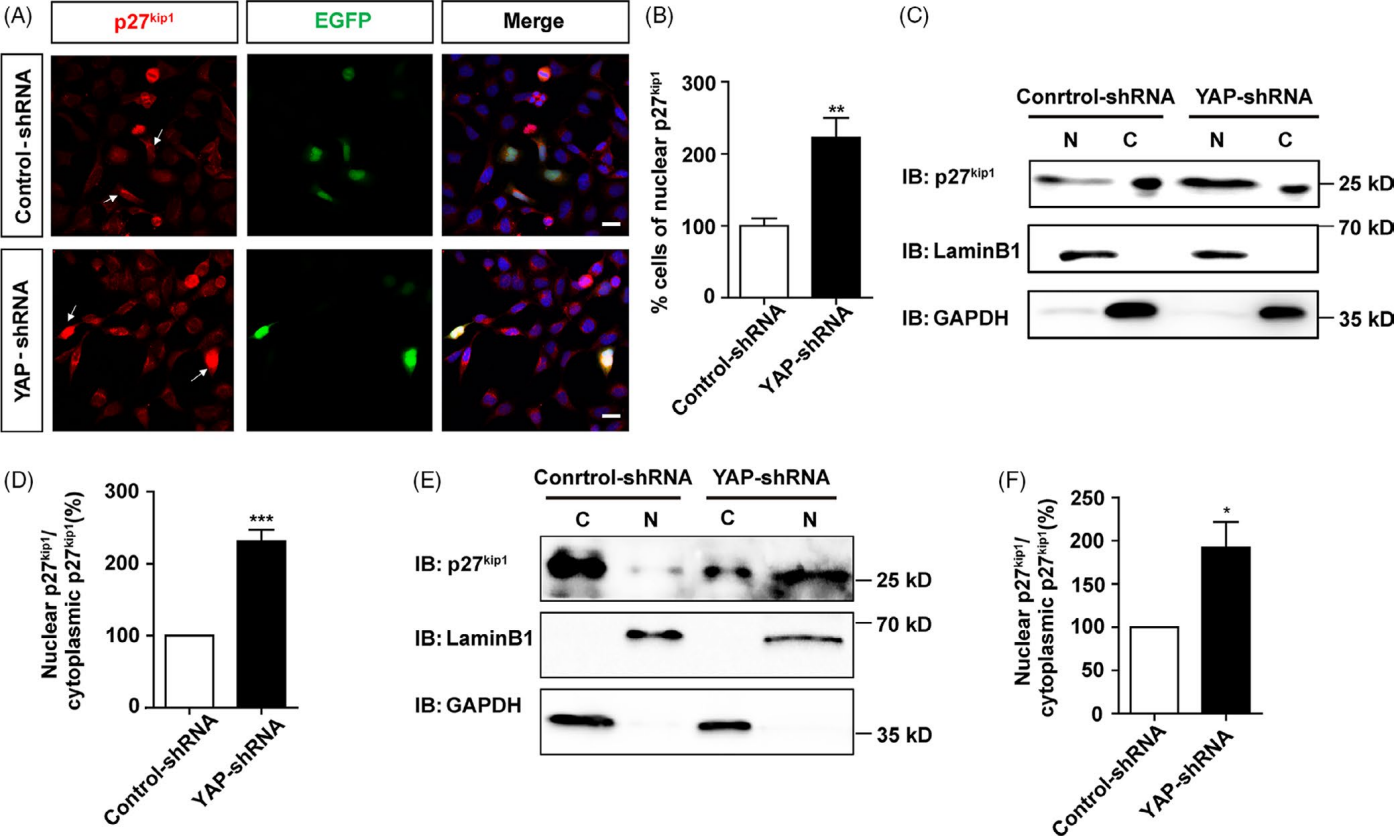
Knock‐down of YAP increased the nuclear location of p27^Kip1^ in SH‐SY5Y cells. A, Immunostaining analysis of p27^Kip1^ (red) in SH‐SY5Y cells co‐transfected with control‐shRNA, pEGFP or YAP‐shRNA, pEGFP constructs for 48‐72 h. Arrows indicated less nuclear p27^Kip1^‐positive cells in control cell groups, and more nuclear p27^Kip1^‐positive cells in YAP knock‐down cell groups. B, Quantitative analysis of the percentage of nuclear p27^Kip1^‐positive cells over EGFP^+^ cells as shown in A (n = 15). C, Representative Western blot image of the nuclear and cytoplasmic expression of p27^Kip1^ in SH‐SY5Y cells transfected with control‐shRNA or YAP‐shRNA for 48‐72 h. D, Quantitative analysis of the nucleus/cytoplasm ratio of p27^Kip1^ in C (n = 3). E, Representative Western blot image of the nuclear and cytoplasmic expression of p27^Kip1^ in SK‐N‐SH cells transfected with control‐shRNA or YAP‐shRNA for 48‐72 h. F, Quantitative analysis of the nucleus/cytoplasm ratio of p27^Kip1^ in E (n = 6). Scale bars, 20 μm. Data were mean ± SEM. **P < .01, ***P < .001

Since the subcellular localization of p27^Kip1^ can affect cell proliferation,^29^, ^30^ we next examined whether YAP enhanced cell proliferation by controlling the nuclear/cytoplasmic location of p27^Kip1^. As shown in Figure 3A,B, knock‐down of YAP in SH‐SY5Y cells significantly increased the percentage of nuclear p27^Kip1^‐positive cells, compared with control‐shRNA construct, implying that YAP knock‐down enhanced the nuclear accumulation of p27^Kip1^. Furthermore, the nuclear and cytoplasmic proteins were dissociated and collected by using the nuclear‐cytosol extraction kit, and Western blot showed that knock‐down of YAP significantly increased the nuclear location and nucleus/cytoplasm ratio of p27^Kip1^, both in SH‐SY5Y cells and in SK‐N‐SH cells, compared to control cells (Figure 3C‐F). As phosphorylation of p27^kip1^ at Ser10 facilitates its binding to the carrier protein for nuclear export and subsequent transport from the nucleus to the cytoplasm,^32^ we next tested the level of p‐p27^kip1^‐Ser10 in YAP‐downregulated SH‐SY5Y cells. As shown in Figure S3A,B, YAP knock‐down significantly decreased the level of p‐p27^kip1^‐Ser10, indicating more p27^kip1^ molecules will accumulate in the nucleus. These results suggest that knock‐down of YAP increases the nuclear location of p27^Kip1^, which may inhibit the proliferation of SH‐SY5Y cells.

### 3.4 Activation of YAP promoted serum‐induced cell proliferation via decreasing the nuclear location of p27^Kip1^

**Figure 4:**
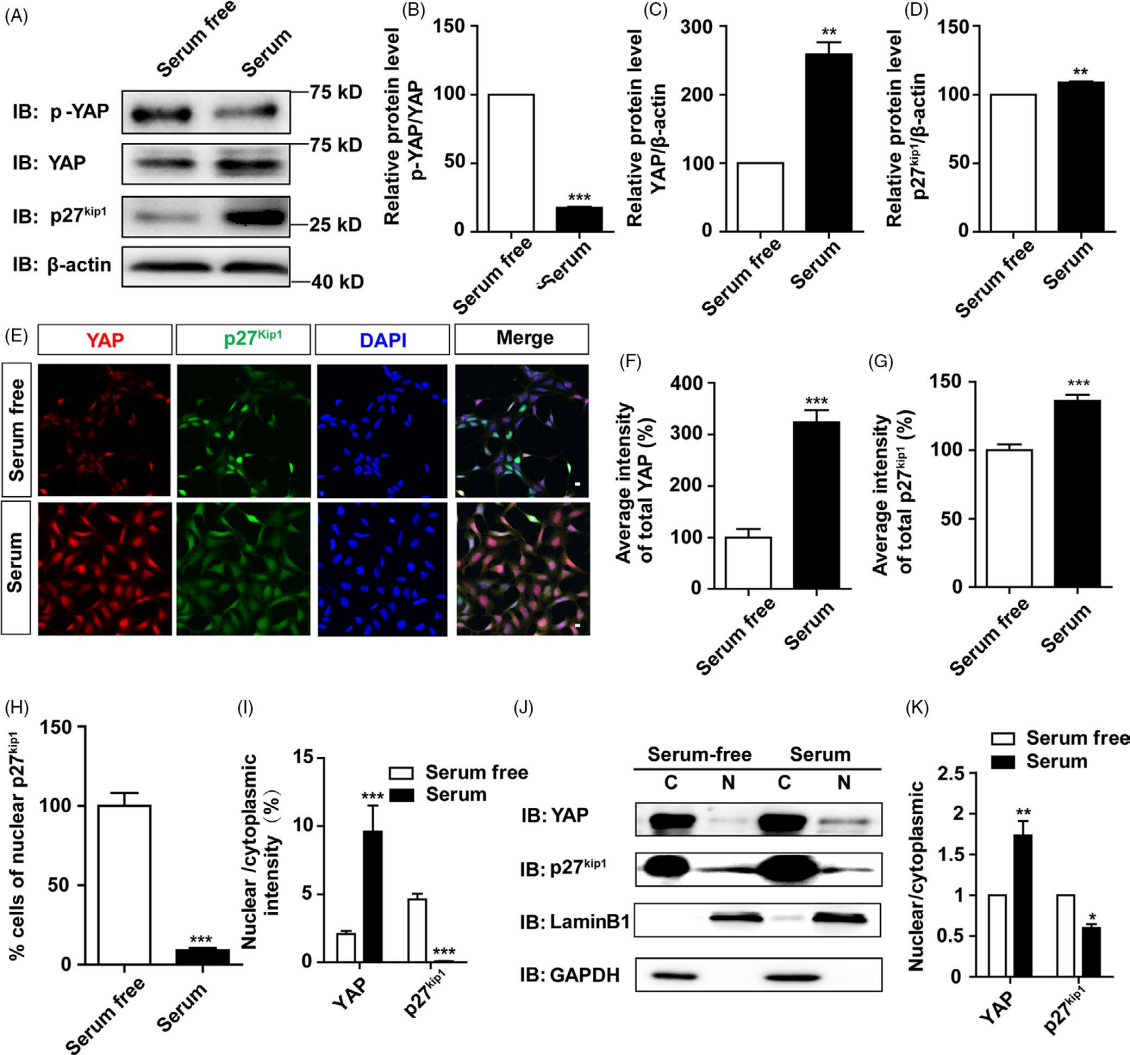
Serum treatment increased the nuclear location of YAP and decreased the nuclear location of p27^Kip1^. A, Representative Western blot image of the expression of p‐YAP, YAP and p27^Kip1^ in SH‐SY5Y cells treated with serum for 24 h. B‐D, Quantification of p‐YAP/YAP (B), YAP (C) and p27^Kip1^ (D) expression as shown in A (n = 3). (E) Double immunostaining of YAP (red) and p27^Kip1^ (green) in SH‐SY5Y cells treated with serum for 24 h. F and G, Quantification of the average intensity of YAP and p27^Kip1^ as shown in E (n = 15). H, Quantification of the percentage of nuclear p27^Kip1^‐positive cells over total cells as shown in E (n = 15). I, Quantitative analysis of the nuclear/cytoplasm ratio of YAP and p27^Kip1^ as shown in E (n = 15). J, Representative Western blot image of the nuclear and cytoplasmic expression of YAP and p27^Kip1^ in SH‐SY5Y cells treated with serum for 24 h. K, Quantitative analysis of the nucleus/cytoplasm ratio of YAP and p27^Kip1^ as shown in J (n = 3). Scale bars, 20 μm. Data were mean ± SEM. *P < .05, **P < .01, ***P < .001

Since several previous studies have shown that serum treatment can increase YAP expression, promote the nuclear translocation of YAP and activate YAP,^9^, ^36^ thus we next tested whether activation of YAP by serum can promote cell proliferation via decreasing the nuclear location of p27^Kip1^. As shown in Figure 4A‐D, Western blot showed that serum treatment significantly decreased p‐YAP level and increased YAP level, suggesting that serum indeed activated YAP, meanwhile, serum treatment also significantly increased p27^Kip1^ level in SH‐SY5Y cells. Interestingly, double immunostaining analysis of p27^Kip1^ and YAP in SH‐SY5Y cells treated by serum showed that YAP expression was effectively upregulated, and the average intensity of p27^Kip1^ increased as well (Figure 4E‐G). However, the percentage of nuclear p27^Kip1^‐positive cells was significantly decreased by serum treatment, compared to control cells cultured in serum‐free media (Figure 4H).

Similarly, we further quantified the nuclear and cytoplasmic location of p27^Kip1^ by immunostaining and nuclear‐cytosol fractionation. As expected, immunostaining showed that the nucleus/cytoplasm ratio of YAP was significantly increased, whereas the nucleus/cytoplasm ratio of p27^Kip1^ was significantly decreased by serum treatment (Figure 4I). Again, the nuclear‐cytosol dissociation experiment showed increased nuclear location and nucleus/cytoplasm ratio of YAP and reduced nuclear location and nucleus/cytoplasm ratio of p27^Kip1^ by serum treatment, compared to cells cultured in serum‐free media (Figure 4J,K). In addition, we tested the level of p‐p27^kip1^‐Ser10 in YAP‐overexpressed SH‐SY5Y cells. As shown in Figure S3C,D, YAP overexpression significantly increased the level of p‐p27^kip1^‐Ser10, indicating that less p27^kip1^ molecules will stay in the nucleus. Taken together, these results strongly suggest that activation of YAP decreases the nuclear location of p27^Kip1^ in SH‐SY5Y cells, which may promote cell proliferation.

**Figure 5:**
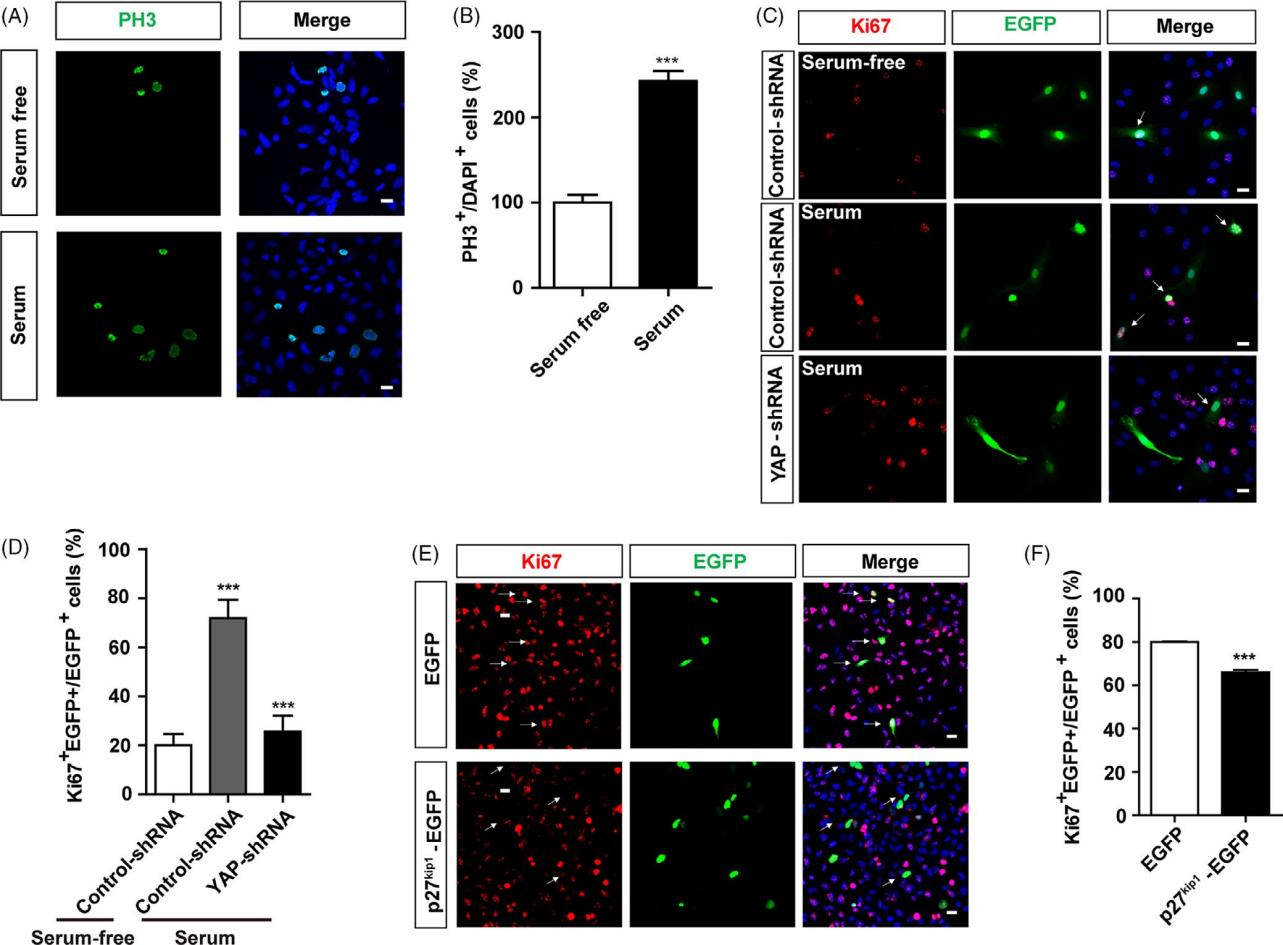
Activation of YAP mediated serum‐induced cell proliferation via decreasing the nuclear location of p27^Kip1^. A, Immunostaining analysis of PH3 (green) in SH‐SY5Y cells treated with serum‐free or serum‐containing culture media for 24 h. B, Quantitative analysis of the percentages of PH3‐positive cells over total SH‐SY5Y cells as shown in A (n = 15). C, Immunostaining analysis of Ki67 (red) in SH‐SY5Y cells co‐transfected with control‐shRNA, pEGFP or YAP‐shRNA, pEGFP constructs for 48 h. Arrows indicated that most of the cells co‐transfected with control‐shRNA and EGFP showed Ki67‐positive signals, and large of the cells co‐transfected with control‐shRNA and EGFP without serum or YAP‐shRNA and EGFP with serum showed Ki67‐negative signals. D, Quantitative analysis of the percentages of Ki67^+^ and EGFP^+^ cells over EGFP^+^ SH‐SY5Y cells as shown in C (n = 15). E, Immunostaining of Ki67 (red) in SH‐SY5Y cells transfected with pEGFP or pEGFP‐p27^kip1^ plasmids for 48‐72 h. Arrows indicated that most of the cells transfected with EGFP showed Ki67‐positive signals, and cells transfected with p27^Kip1^‐EGFP showed Ki67 negative signals largely. F, Analysis of the percentage of Ki67^+^ and EGFP^+^ cells over EGFP^+^ cells as shown in E (n = 15). Scale bars, 20 μm. Data were mean ± SEM. ***P < .001

We next examined whether the decrease of nuclear location of p27 ^Kip1^ by YAP activation was required for serum‐induced cell proliferation. As shown in Figure 5A,B, serum treatment significantly increased the proliferation of SH‐SY5Y cells, and knock‐down of YAP inhibited the proliferation of SH‐SY5Y induced by serum, suggesting that YAP activation was required for serum‐induced proliferation for SH‐SY5Y cells (Figure 5C,D). Interestingly, overexpression of p27^Kip1^‐EGFP in SH‐SY5Y cells cultured with 10% serum (under YAP activation condition) indeed significantly decreased the proliferation of SH‐SY5Y cells, suggesting that overexpression of p27^Kip1^ reduced the proliferation of SH‐SY5Y cells under YAP activation conditions (Figure 5E,F, Figure S4). Moreover, as we see, overexpressed p27^Kip1^ was mostly expressed in the nucleus (Figure S4), which reminded us that nuclear location of p27^Kip1^ may be the real reason that inhibits cell proliferation. Taken together, these results suggest that YAP may promote cell proliferation via decreasing the nuclear location of p27^Kip1^.

### 3.5 Overexpression of YAP restored the nuclear location of p27^Kip1^ in serum‐starved SH‐SY5Y cells

**Figure 6:**
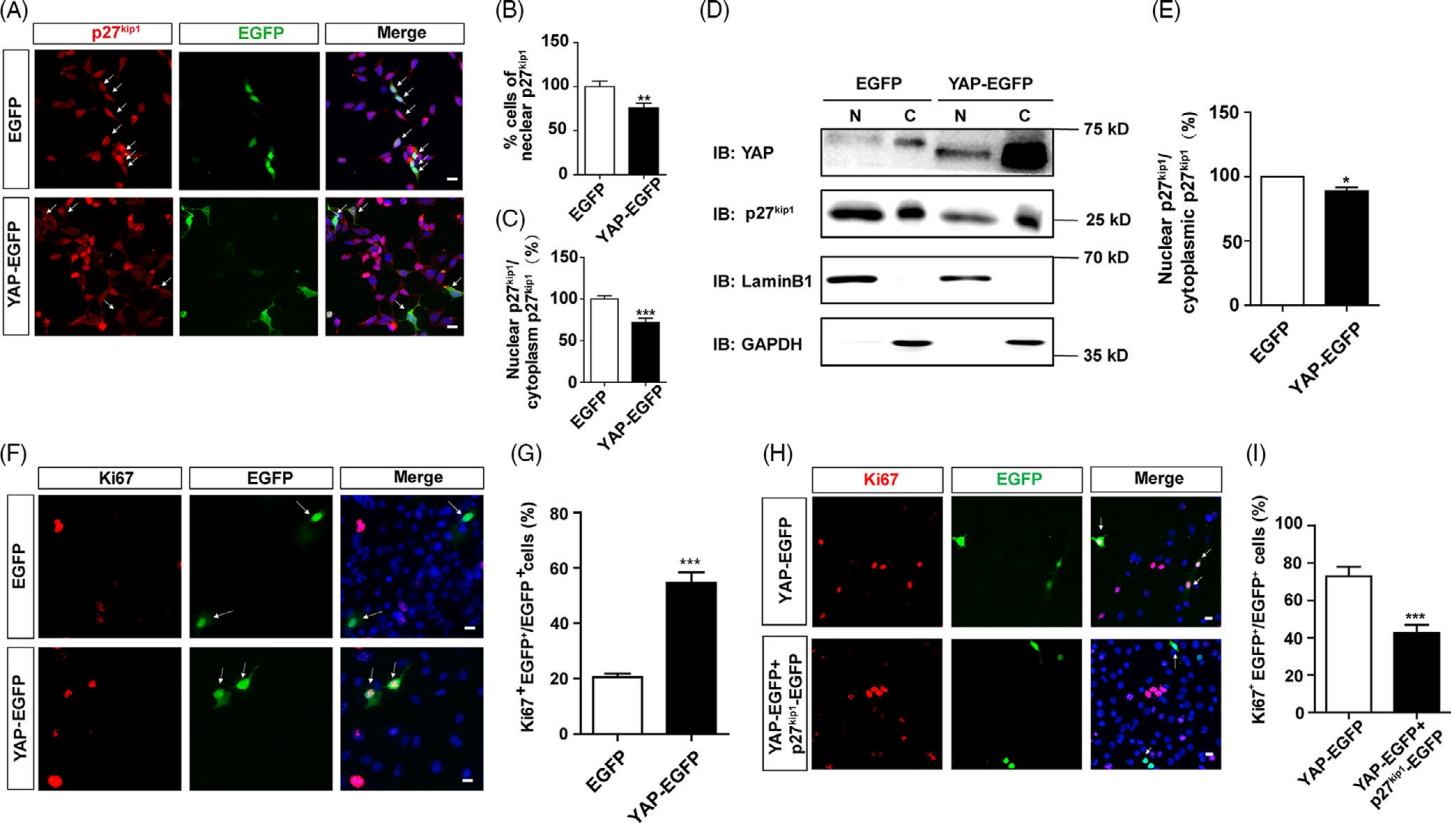
Overexpression of YAP restored the nuclear location of p27^Kip1^ in serum‐starved SH‐SY5Y cells. A, Immunostaining analysis of p27^Kip1^ (red) in SH‐SY5Y cells transfected with EGFP or YAP‐EGFP cultured in serum‐free media for 48 h. Arrows indicated that part of the cells transfected with EGFP‐exhibited nuclear distribution of p27^Kip1^, whereas cells transfected with YAP‐EGFP mostly showed the cytoplasmic location of p27^Kip1^. B and C, Quantification of the percentage of nuclear p27^Kip1^‐positive cells over total cells and quantitative analysis of the nucleus/cytoplasm ratio of p27^Kip1^ as shown in A (n = 15). D, Representative Western blot image of the nuclear and cytoplasmic expression of YAP and p27^Kip1^ in SH‐SY5Y cells transfected with EGFP or YAP‐EGFP cultured in serum‐free media for 48 h. E, Quantitative analysis of the nucleus/cytoplasm ratio of p27^Kip1^ as shown in D (n = 3). F, Immunostaining of Ki67 (red) in SH‐SY5Y cells. Cells were transfected with EGFP or YAP‐EGFP for 48 h following 24 h cultured in serum‐free media. Arrows indicated EGFP‐positive cells that with or without co‐localization of Ki67. G, Quantification of the percentage of Ki67^+^ and EGFP^+^ cells over EGFP^+^ cells as shown in F (n = 15). H, Immunostaining of Ki67 (red) in SH‐SY5Y cells transfected with pEGFP‐YAP or pEGFP‐YAP plus pEGFP‐p27^Kip1^ plasmids in serum‐free media for 48 h. Arrows indicated that large amount of the cells transfected with YAP‐EGFP showed Ki67‐positive signals, and cells transfected with YAP‐EGFP and p27^Kip1^‐EGFP showed less Ki67‐positive signals. I, Analysis of the percentage of Ki67^+^ and EGFP^+^ cells over EGFP^+^ cells as shown in H (n = 15). Scale bars, 20 μm. Data were mean ± SEM. *P < .05, **P < .01, ***P < .001

To examine whether YAP activation is sufficient to restore the nuclear location of p27^Kip1^ and thereby promotes cell proliferation, we performed YAP overexpression experiments in SH‐SY5Y cells cultured in serum‐free media. As shown in Figure 6A‐C, SH‐SY5Y cells cultured in serum‐free media were transfected with YAP‐EGFP or EGFP constructs, and immunostaining analysis of p27^Kip1^ in these transfected cells showed that the percentage of nuclear p27^Kip1^ was significantly decreased, and the nucleus/cytoplasm ratio of p27^Kip1^ was significantly decreased in YAP‐EGFP over‐expressing SH‐SY5Y cells, compared with the EGFP control. Furthermore, the nuclear‐cytosol dissociation experiments also showed that overexpression of YAP significantly decreased the nuclear location and nucleus/cytoplasm ratio of p27^Kip1^, compared with EGFP control (Figure 6D,E). Furthermore, the overexpression of YAP significantly promoted cell proliferation of SH‐SY5Y cells, compared with EGFP control (Figure 6F,G, Figure S5). Finally, overexpression of YAP together with p27^Kip1^ in SH‐SY5Y cells cultured in serum‐free media (YAP inactivation condition) decreased cell proliferation, compared with YAP‐EGFP alone (Figure 6H,I, Figure S6A‐C). Taken together, these results suggest overexpression of YAP is sufficient to restore the nuclear translocation of p27^Kip1^ in serum‐free media, which promotes cell proliferation of SH‐SY5Y cells.

### 3.6 YAP negatively regulated the nuclear location of p27^Kip1^ in SH‐SY5Y cells through Akt

**Figure 7:**
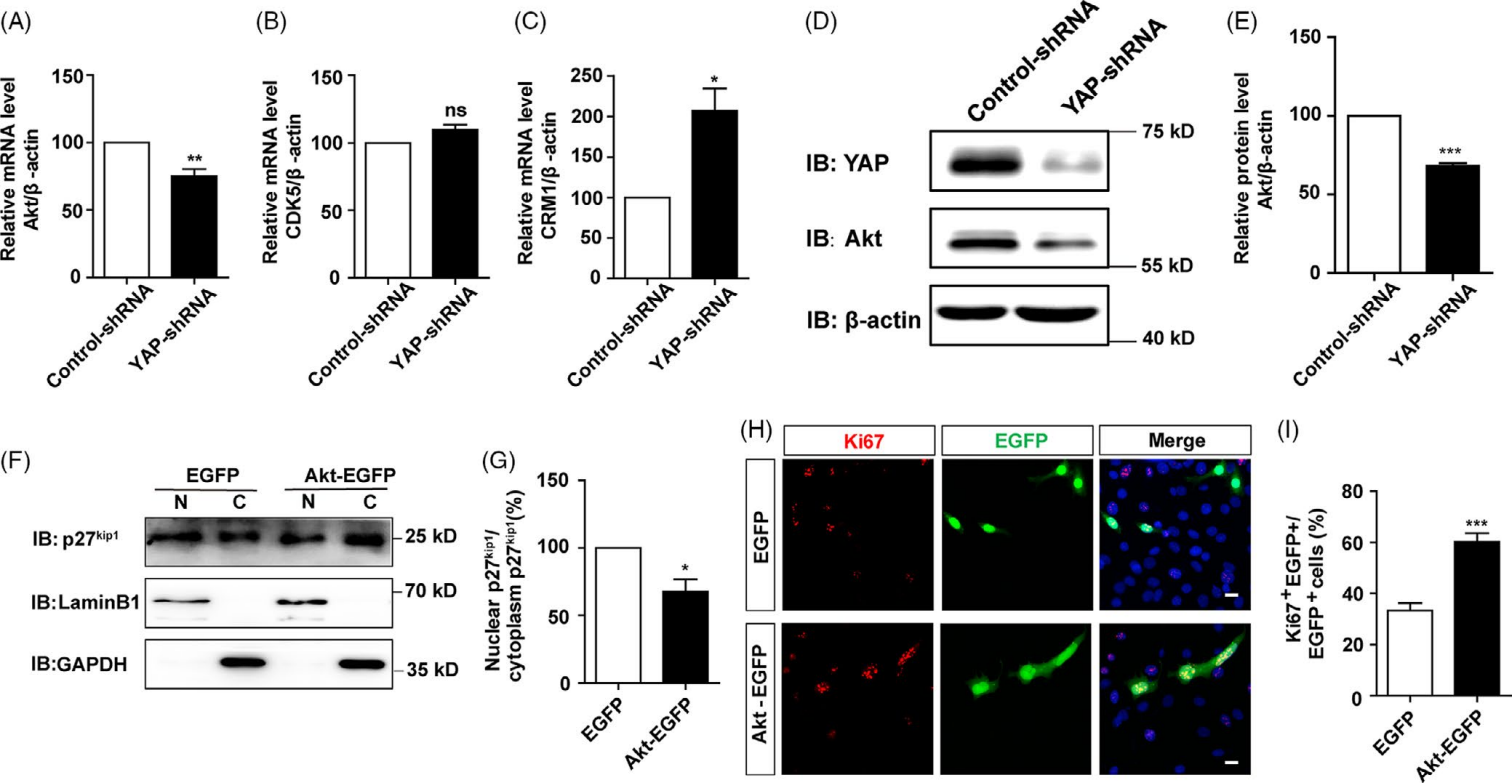
YAP negatively regulated the nuclear location of p27^Kip1^ in SH‐SY5Y cells through Akt. A‐C, qPCR analysis of the mRNA level of Akt, CDK5 and CRM1 in YAP control and knock‐down cells (n = 3). D, Representative Western blot image of the expression of Akt in SH‐SY5Y cells transfected with vectors encoding control‐shRNA or YAP‐shRNA for 48‐72 h. E, Quantification of Akt expression as shown in D (n = 3). F, Representative Western blot image of the nuclear and cytoplasmic expression of p27^Kip1^ in SH‐SY5Y cells transfected with control or Akt following YAP‐shRNA transfection for 24 h. G, Quantitative analysis of the nucleus/cytoplasm ratio of p27^Kip1^ as shown in F (n = 5). H, Immunostaining of Ki67 (red) in SH‐SY5Y cells transfected with control or Akt following serum‐free treatment for 24 h. I, Quantification of the percentage of Ki67^+^ and EGFP^+^ cells over EGFP^+^ cells as shown in H (n = 15). Scale bars, 20 μm. Data were mean ± SEM. *P < .05, **P < .01, ***P < .001

How does YAP regulate the subcellular location of p27^Kip1^ in SH‐SY5Y cells? Previous studies have shown that Akt can promote the nuclear export of p27^Kip1^ and hinder nuclear import of p27^Kip1^ through phosphorylation^37^; thus, we next examined whether YAP regulated the subcellular location of p27^Kip1^ through controlling gene transcription of Akt. Interestingly, as shown in Figure 7A‐C, real‐time PCR results indeed showed that Akt mRNA level was significantly reduced in YAP knock‐down SH‐SY5Y cells, compared with control cells, whereas mRNA level of CDK5 (another kinase that promotes the nuclear export of p27^Kip1^) did not display significant change in YAP knock‐down SH‐SY5Y cells, and CRM1, a carrier protein that responsible for the nuclear export of p27^Kip1^, showed increased mRNA expression. Furthermore, Western blot also detected decreased level of Akt protein in YAP knock‐down SH‐SY5Y cells, compared with control cells (Figure 7D,E), suggesting Akt might be a downstream target of YAP. To further know whether Akt is sufficient to induce the nuclear export of p27^Kip1^ in cells with low YAP activity, Akt was overexpressed in YAP‐shRNA transfected SH‐SY5Y cells. The nuclear‐cytosol dissociation experiment showed that overexpression of Akt significantly decreased the nuclear location and nucleus/cytoplasm ratio of p27^Kip1^, compared with EGFP control (Figure 7F,G, Figure S7A,B). Finally, overexpression of Akt significantly promoted the proliferation of SH‐SY5Y cells, compared with EGFP control (Figure 7H,I). These results strongly suggest that YAP promotes the cell proliferation of SH‐SY5Y cells by negatively regulating the nuclear location of p27^Kip1^ through modulating the Akt expression.

## 4 DISCUSSION

**Figure 8:**
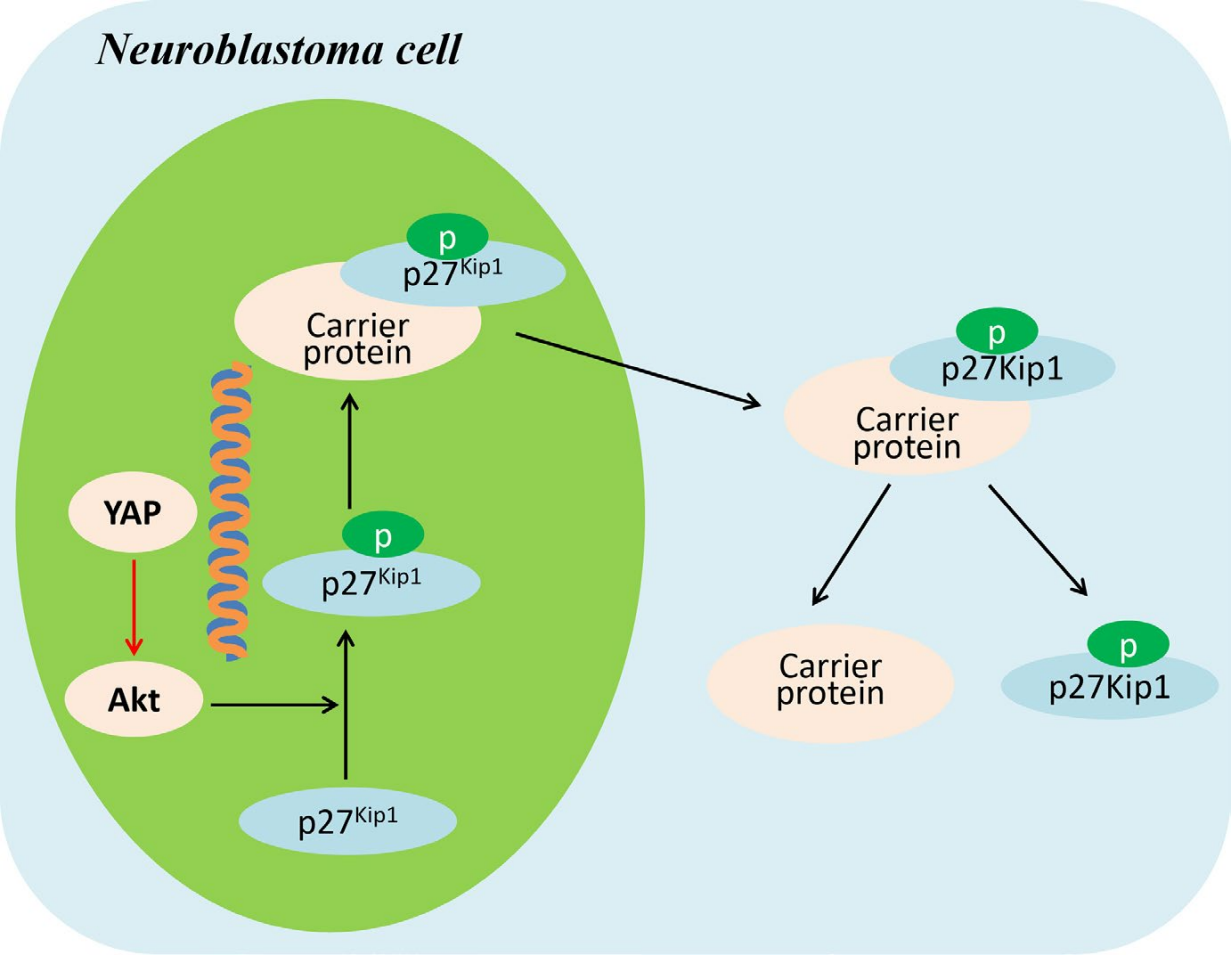
Working model of YAP functions in the proliferation of neuroblastoma cells. Mitogenic factors such as serum promote the nuclear translocation of YAP, which initiates the transcription of target genes such as Akt. Furthermore, upregulation of Akt expression promotes the nuclear export of p27^Kip1^ and decreases the nuclear location of p27^Kip1^, which enhances proliferation

Here, we present evidence for YAP function in neuroblastoma proliferation and propose a working model, as depicted in Figure 8. In this model, mitogenic factor such as serum promotes the nuclear translocation of YAP, which initiates the transcription of target genes such as Akt. Furthermore, upregulation of Akt expression promotes the nuclear export of p27^Kip1^ and decreases the nuclear location of p27^Kip1^, which result in the enhanced proliferation of neuroblastoma cells.

YAP is highly expressed in human glioma specimens, compared to non‐tumour human brain tissues.^38^ Immunohistochemical staining shows that the expression level of YAP is significantly higher in the malignant neuroblastoma tissues of human, compared to the peritumoral tissues.^13^ Moreover, the high expression of YAP in neuroblastoma is correlated with tumour grade of neuroblastoma.^13^ Consistent with these studies, our results showed that YAP was abundantly expressed in different glioma cell lines, and the expression level of YAP was most enriched in the neuroblastoma cell line, SH‐SY5Y and mainly displayed the nuclear location. The nuclear location of YAP implies that YAP may regulate the proliferation of neuroblastoma. Cell proliferation is a vital process for normal development of our body and brain. For a long time, YAP is known to be an important regulator of cell proliferation, regardless of normal cell or tumour cells.^9^, ^39^, ^40^, ^41^, ^42^, ^43^, ^44^ Moreover, YAP can prevent reactive astrogliosis.^9^ Consistent with these previous studies, our results also suggest that YAP promotes the proliferation of neuroblastoma cells based on several proliferation assays. However, it remains unclear how YAP regulates the proliferation of neuroblastoma cells.

There are evidences revealing that YAP can modulate tumour cell proliferation through regulating the expression of cell cycle proteins, such as p27^Kip1^, p21^45^ and cyclinD1.^25^, ^28^, ^43^ p27^Kip1^ is a cyclin‐dependent kinases inhibitor that involved in the pathogenesis of neuroblastoma. Decreased transcription level of p27^Kip1^ increases human susceptibility to neuroblastoma.^22^ Several previous studies have shown that YAP can regulate the proliferation of tumour cells by modulating the total expression level of p27^Kip1^.^24^, ^25^, ^26^, ^27^, ^46^ However, the subcellular location of p27^Kip1^ protein plays the critical roles in the regulation of cell proliferation and the nuclear‐located of p27^Kip1^ exerts its anti‐proliferative action.^29^, ^30^, ^31^, ^37^ In our present study, several lines of evidence suggest that YAP promotes the proliferation of neuroblastoma through controlling the subcellular location of p27^Kip1^. First, YAP knock‐down increased the nuclear location of p27^Kip1^ in the SH‐SY5Y cells. Second, YAP activation decreased the nuclear location of p27^Kip1^ in the SH‐SY5Y cells and SK‐N‐SH cells. Third, overexpression of YAP restored the nuclear location of p27^Kip1^ in serum‐starved SH‐SY5Y cells. The view that YAP regulates the subcellular location of p27^Kip1^ is also in line with recent findings. For example, YAP can directly promote the transcription of Skp2 (a subunit of E3 ubiquitin ligase), reduce the total expression level of p27^Kip1^ and accelerate the proliferation of human breast cancer cells.^28^ Skp2 degrades p27^Kip1^ mainly in the nucleus,^37^ so YAP mainly promotes the degradation of p27^Kip1^ in the nucleus, and reduces nuclear p27^Kip1^, thereby accelerating cell proliferation. Further studies will be performed to test these possibilities in future. In our study, surprisingly, we found that the total level of p27^kip1^ was decreased when YAP was knock‐down (Figure S8). Although decreased level of total p27^kip1^ would promote the proliferation of SH‐SY5Y cells according to traditional concepts, the effects of increased nuclear accumulation of p27^kip1^ might be more than the effects of decreased level of total p27^kip1^, which may be the main reason that caused reduced proliferation of SH‐SY5Y cells by YAP knock‐down. This view is supported by the publications that showing nuclear p27^kip1^ inhibits cell proliferation, and cytoplasmic p27^kip1^ promotes cell proliferation.^29^, ^30^, ^31^, ^37^ To know why knock‐down of YAP led to a decrease in total expression level of p27^kip1^, further studies are needed.

We also noticed that serum treatment activated YAP and increased p27^kip1^ level in SH‐SY5Y cells (Figure 4); however, when YAP was overexpressed, the p27^kip1^ level in both nucleus and cytoplasm seemed significantly reduced (Figure 6). This may be the reason that overexpression of YAP is a treatment that may be different from serum activation. Serum treatment just activated the endogenous YAP and promoted the nuclear translocation of YAP in cells; however, YAP overexpression introduced the exogenous YAP on the basis of endogenous YAP, and YAP was expressed globally in the nucleus and cytoplasm, which might cause the difference in the nuclear and cytoplasmic level of p27^kip1^.

How YAP transcriptionally regulates the expression of Akt? Actually, at present, there are no reports about how YAP regulates the expression of Akt at the transcription level; however, there are some articles reported the relationship between YAP and Akt.^12^, ^47^, ^48^, ^49^, ^50^, ^51^ For example, knock‐down of YAP can repress the activation of Akt in colorectal cancer cells,^47^ silencing of YAP expression markedly attenuates AMOT‑induced expression of p‐Akt,^48^ YAP induces IGF2‐mediated activation of Akt in medulloblastoma.^12^ These results suggest that Akt might be the downstream target of YAP. In our study, serum starvation decreased the level of p‐Akt‐Thr308 in SH‐SY5Y cells significantly (Figure S9), indicating that the change of p27^kip1^ distribution might be dependent on Akt phosphorylation. Interestingly, YAP could also serve as a downstream target of Akt signalling.^50^, ^51^ YAP is identified as a substrate of Akt and can be phosphorylated by Akt, which attenuates p73‐mediated apoptosis.^50^ The Akt inhibitor Akt VIII decreased p‐YAP level and increased YAP level.^51^ Therefore, there may be a YAP‐Akt‐YAP feedback loop existing in the regulation of neuroblastoma proliferation.

Moreover, as we know, YAP is a transcriptional co‐activator, it has no DNA‐binding domain and cannot bind to DNA directly.^52^ Therefore, the transcriptional expression of target genes regulated by YAP requires DNA‐binding transcription factors. The transcription factor of TEAD family member is the major binding molecule of YAP.^5^, ^33^, ^53^, ^54^, ^55^ The hydrogen bond formed by YAP S94 and TEAD1 Y406 was critical for YAP/TEAD‐mediated tissue development and homeostasis.^56^ Actually, the functions of YAP are dependent on TEAD at most conditions. There are several reports showing that YAP promotes cell proliferation through TEAD.^5^, ^57^, ^58^, ^59^, ^60^ Therefore, there is great possibility that the regulation of Akt by YAP is dependent on TEAD family members. Nevertheless, to know whether the regulation of Akt by YAP is dependent on TEAD family members exactly, further experiments involving YAP‐TEAD inhibitors are needed.

The nuclear p27^Kip1^ can be regulated by nuclear export,^29^, ^30^, ^31^, ^32^, ^61^ nuclear import^62^, ^63^ and nuclear degradation.^37^, ^64^, ^65^ Currently, the nuclear export of p27^Kip1^ is mainly determined by phosphorylation of p27^Kip1^ on serine.^10^ Phosphorylation of p27^Kip1^ on serine^10^ by Akt, CDK5 and KIS (Kinase Interacting Stathmin) in the early G1 phase is necessary for its binding to a carrier protein CRM1 for nuclear export, which promotes cell proliferation subsequently.^29^, ^30^, ^31^, ^32^ However, in our study, we found that the mRNA level of CDK5 has no significant change, and CRM1 exhibited increased mRNA level in YAP knock‐down cells, indicating that they are not direct target genes of YAP, and accumulation of p27^Kip1^ in the nucleus of YAP knock‐down cells may not be mediated by CDK5 or CRM1. In the early G1 phase, Thr157 and Thr198 of p27^Kip1^ are phosphorylated by Akt, p90RSK1 (p90 ribosomal protein S6 kinases), SGK (serum and glucocorticoid‐inducible kinase), AMPK and PIM (although this phosphorylation is relatively rare), which will prevent the nuclear transfer of p27^Kip1^.^62^ Moreover, Akt induces phosphorylation of Thr157 and Thr198 to form a recognition motif for 14‐3‐3 protein to prevent nuclear translocation of p27^Kip1^.^63^ Thus, Akt might also implicate in the nuclear and cytoplasmic distribution of p27^Kip1^. Interestingly, in our study, we found that the mRNA and protein level of Akt were both decreased by YAP knock‐down, overexpression of Akt increased the cytoplasmic location of p27^kip1^ and partially rescued the effects induced by YAP inactivation. Therefore, Akt may be the downstream target of YAP, controlling the nuclear/cytoplasmic distribution of p27^kip1^ and cell proliferation. Further studies will be performed to examine how YAP regulates the expression of Akt.

So far, no evidence has shown that YAP‐Akt‐p27^kip1^ signalling is involved in the proliferation of neuroblastoma in vivo, as well as YAP‐Akt, Akt‐p27^kip1^ and YAP‐p27^kip1^ signalling. However, there are in vivo results suggesting that YAP, Akt and p27^kip1^ are related to neuroblastoma.^13^, ^22^, ^66^ YAP is significantly higher in the malignant neuroblastoma tissues of human, compared to the peritumoral tissues.^13^ Inhibition of the Akt signalling pathway shows therapeutic efficacy in neuroblastoma xenografts in vivo.^66^ Decrease of transcript levels of p27^Kip1^ increases human susceptibility to neuroblastoma.^22^

In summary, our results indicate that YAP promotes proliferation of neuroblastoma cells through decreasing the nuclear location of p27^Kip1^ mediated by Akt. Disruption of the balance of p27^Kip1^ distribution between nuclear and cytoplasmic distribution by YAP activation will influence cell cycle and cell proliferation. Our study provides a new mechanism for the proliferation of neuroblastoma cells, and suggest that nuclear p27^Kip1^ entrapment may be a potential therapeutic strategy for anti‐proliferation in neural tumour cells.

## CONFLICTS OF INTEREST

The authors declare no potential conflicts of interest.

## AUTHOR CONTRIBUTIONS

Zhihui Huang and Xingxing Xu contributed to the conception and design of the study. Xiya Shen did most of Western blot and immunofluorescent staining experiments. Xingxing Xu was mainly responsible for the cell culture experiments, and they both contributed to the acquisition and interpretation of the data. Changnan Xie helped to do Western blot and organize the pictures. Wenjin Feng drew the working model of YAP functions in neuroblastoma cell proliferation. Huitao Liu, Jingjing Zhang, Danlu Yang, Ling Wang, Wenjin Feng, Leilei Du, Lina Xuan, Chaobo Meng, Qian Wu, Haitao Zhang and Wei Wang helped to extract the plasmids and analyse some of the staining data. Xingxing Xu wrote the manuscript. Xingxing Xu, Ying Wang, Tian Xie and Zhihui Huang contributed to revising the original text.

## Data Availability

The data that support the findings of this study are available from the corresponding author upon reasonable request.

## References

[1] Yu FX , Zhao B , Guan KL . Hippo pathway in organ size control, tissue homeostasis, and cancer. Cell. 2015;163(4):811‐828. 26544935 10.1016/j.cell.2015.10.044 PMC4638384

[2] Pan D . The hippo signaling pathway in development and cancer. Dev Cell. 2010;19(4):491‐505. 20951342 10.1016/j.devcel.2010.09.011 PMC3124840

[3] Chung H , Lee BK , Uprety N , Shen W , Lee J , Kim J . Yap1 is dispensable for self‐renewal but required for proper differentiation of mouse embryonic stem (ES) cells. EMBO Rep. 2016;17(4):519‐529. 26917425 10.15252/embr.201540933 PMC4818770

[4] Tang Y , Feinberg T , Keller ET , Li XY , Weiss SJ . Snail/Slug binding interactions with YAP/TAZ control skeletal stem cell self‐renewal and differentiation. Nat Cell Biol. 2016;18(9):917‐929. 27479603 10.1038/ncb3394 PMC5007193

[5] Chen L , Chan SW , Zhang X , et al. Structural basis of YAP recognition by TEAD4 in the hippo pathway. Genes Dev. 2010;24(3):290‐300. 20123908 10.1101/gad.1865310 PMC2811830

[6] Ahmed AA , Mohamed AD , Gener M , Li W , Taboada E . YAP and the Hippo pathway in pediatric cancer. Mol Cell Oncol. 2017;4(3):e1295127. 28616573 10.1080/23723556.2017.1295127 PMC5462521

[7] Zanconato F , Cordenonsi M , Piccolo S . YAP/TAZ at the roots of cancer. Cancer Cell. 2016;29(6):783‐803. 27300434 10.1016/j.ccell.2016.05.005 PMC6186419

[8] Huang Z , Wang Y , Hu G , Zhou J , Mei L , Xiong WCYAP . YAP is a critical inducer of SOCS3, preventing reactive astrogliosis. Cereb Cortex. 2016;26(5):2299‐2310. 26679195 10.1093/cercor/bhv292 PMC4830299

[9] Huang Z , Hu J , Pan J , et al. YAP stabilizes SMAD1 and promotes BMP2‐induced neocortical astrocytic differentiation. Development. 2016;143(13):2398‐2409. 27381227 10.1242/dev.130658 PMC4958318

[10] Huang Z , Sun D , Hu JX , et al. Neogenin promotes BMP2 activation of YAP and Smad1 and enhances astrocytic differentiation in developing mouse neocortex. J Neurosci. 2016;36(21):5833‐5849. 27225772 10.1523/JNEUROSCI.4487-15.2016 PMC4879200

[11] Fernandez LA , Northcott PA , Dalton J , et al. YAP1 is amplified and up‐regulated in hedgehog‐associated medulloblastomas and mediates Sonic hedgehog‐driven neural precursor proliferation. Genes Dev. 2009;23(23):2729‐2741. 19952108 10.1101/gad.1824509 PMC2788333

[12] Fernandez LA , Squatrito M , Northcott P , et al. Oncogenic YAP promotes radioresistance and genomic instability in medulloblastoma through IGF2‐mediated Akt activation. Oncogene. 2012;31(15):1923‐1937. 21874045 10.1038/onc.2011.379 PMC3583298

[13] Yang C , Tan J , Zhu J , Wang S , Wei G . YAP promotes tumorigenesis and cisplatin resistance in neuroblastoma. Oncotarget. 2017;8(23):37154‐37163. 28415761 10.18632/oncotarget.16209 PMC5514898

[14] Matthay KK , Maris JM , Schleiermacher G , et al. Neuroblastoma. Nat Rev Dis Primers. 2016;2:16078. 27830764 10.1038/nrdp.2016.78

[15] Schramm A , Koster J , Assenov Y , et al. Mutational dynamics between primary and relapse neuroblastomas. Nat Genet. 2015;47(8):872‐877. 26121086 10.1038/ng.3349

[16] Wang Q , Xu Z , An Q , et al. TAZ promotes epithelial to mesenchymal transition via the upregulation of connective tissue growth factor expression in neuroblastoma cells. Mol Med Rep. 2015;11(2):982‐988. 25354978 10.3892/mmr.2014.2818 PMC4262480

[17] Nakayama KI , Nakayama K . Ubiquitin ligases: cell‐cycle control and cancer. Nat Rev Cancer. 2006;6(5):369‐381. 16633365 10.1038/nrc1881

[18] Bergmann E , Wanzel M , Weber A , Shin I , Christiansen H , Eilers M . Expression of P27(KIP1) is prognostic and independent of MYCN amplification in human neuroblastoma. Int J Cancer. 2001;95(3):176‐183. 11307151 10.1002/1097-0215(20010520)95:3<176::aid-ijc1030>3.0.co;2-z

[19] Omura‐Minamisawa M , Diccianni MB , Chang RC , et al. p16/p14(ARF) cell cycle regulatory pathways in primary neuroblastoma: p16 expression is associated with advanced stage disease. Clin Cancer Res. 2001;7(11):3481‐3490. 11705866

[20] Jung H , Shin JH , Park YS , Chang MS . Ankyrin repeat‐rich membrane spanning (ARMS)/Kidins220 scaffold protein regulates neuroblastoma cell proliferation through p21. Mol Cells. 2014;37(12):881‐887. 25410904 10.14348/molcells.2014.0182 PMC4275705

[21] Yogev O , Barker K , Sikka A , et al. p53 loss in MYC‐driven neuroblastoma leads to metabolic adaptations supporting radioresistance. Cancer Res. 2016;76(10):3025‐3035. 27197232 10.1158/0008-5472.CAN-15-1939

[22] Capasso M , McDaniel LD , Cimmino F , et al. The functional variant rs34330 of CDKN1B is associated with risk of neuroblastoma. J Cell Mol Med. 2017;21(12):3224‐3230. 28667701 10.1111/jcmm.13226 PMC5706517

[23] Nakamura Y , Ozaki T , Koseki H , Nakagawara A , Sakiyama S . Accumulation of p27 KIP1 is associated with BMP2‐induced growth arrest and neuronal differentiation of human neuroblastoma‐derived cell lines. Biochem Biophys Res Commun. 2003;307(1):206‐213. 12850001 10.1016/s0006-291x(03)01138-0

[24] Hsueh YJ , Chen HC , Wu SE , Wang TK , Chen JK , Ma DH . Lysophosphatidic acid induces YAP‐promoted proliferation of human corneal endothelial cells via PI3K and ROCK pathways. Mol Ther Methods Clin Dev. 2015;2:15014. 26029725 10.1038/mtm.2015.14 PMC4445000

[25] Zhang S , Chen Q , Liu Q , et al. Hippo signaling suppresses cell ploidy and tumorigenesis through Skp2. Cancer Cell. 2017;31(5):669‐684.e667. 28486106 10.1016/j.ccell.2017.04.004 PMC5863541

[26] Han Y , Tang Z , Zhao Y , Li Q , Wang E . TNFAIP8 regulates Hippo pathway through interacting with LATS1 to promote cell proliferation and invasion in lung cancer. Mol Carcinog. 2018;57(2):159‐166. 28926138 10.1002/mc.22740

[27] Dong Q , Fu L , Zhao Y , et al. Rab11a promotes proliferation and invasion through regulation of YAP in non‐small cell lung cancer. Oncotarget. 2017;8(17):27800‐27811. 28468127 10.18632/oncotarget.15359 PMC5438609

[28] Jang W , Kim T , Koo JS , Kim SK , Lim DS . Mechanical cue‐induced YAP instructs Skp2‐dependent cell cycle exit and oncogenic signaling. EMBO J. 2017;36(17):2510‐2528. 28673931 10.15252/embj.201696089 PMC5579353

[29] Kim JE , Kang TC . Nucleocytoplasmic p27(Kip1) Export Is required for ERK1/2‐mediated reactive astroglial proliferation following status epilepticus. Front Cell Neurosci. 2018;12:152. 29930499 10.3389/fncel.2018.00152 PMC5999727

[30] Li X , Tang X , Jablonska B , Aguirre A , Gallo V , Luskin MB . p27(KIP1) regulates neurogenesis in the rostral migratory stream and olfactory bulb of the postnatal mouse. J Neurosci. 2009;29(9):2902‐2914. 19261886 10.1523/JNEUROSCI.4051-08.2009 PMC3488282

[31] Wang Y , Wang Y , Xiang J , et al. Knockdown of CRM1 inhibits the nuclear export of p27(Kip1) phosphorylated at serine 10 and plays a role in the pathogenesis of epithelial ovarian cancer. Cancer Lett. 2014;343(1):6‐13. 24018641 10.1016/j.canlet.2013.09.002

[32] Ishida N , Hara T , Kamura T , Yoshida M , Nakayama K , Nakayama KI . Phosphorylation of p27Kip1 on serine 10 is required for its binding to CRM1 and nuclear export. J Biol Chem. 2015;290(11):6754. 25770303 10.1074/jbc.A114.100762 PMC4358101

[33] Zhao B , Ye X , Yu J , et al. TEAD mediates YAP‐dependent gene induction and growth control. Genes Dev. 2008;22(14):1962‐1971. 18579750 10.1101/gad.1664408 PMC2492741

[34] Yamada S , Fong MC , Hsiao YW , et al. Impact of renal denervation on atrial arrhythmogenic substrate in ischemic model of heart failure. J Am Heart Assoc. 2018;7(2):e007312. 29358197 10.1161/JAHA.117.007312 PMC5850156

[35] Liu C , Zhai X , Zhao B , Wang Y , Xu Z . Cyclin I‐like (CCNI2) is a cyclin‐dependent kinase 5 (CDK5) activator and is involved in cell cycle regulation. Sci Rep. 2017;7:40979. 28112194 10.1038/srep40979 PMC5256034

[36] Yu FX , Zhao B , Panupinthu N , et al. Regulation of the Hippo‐YAP pathway by G‐protein‐coupled receptor signaling. Cell. 2012;150(4):780‐791. 22863277 10.1016/j.cell.2012.06.037 PMC3433174

[37] Hnit SS , Xie C , Yao M , et al. p27(Kip1) signaling: transcriptional and post‐translational regulation. Int J Biochem Cell Biol. 2015;68:9‐14. 26279144 10.1016/j.biocel.2015.08.005

[38] Zhang Y , Xie P , Wang X , et al. YAP promotes migration and invasion of human glioma cells. J Mol Neurosci. 2018;64(2):262‐272. 29306996 10.1007/s12031-017-1018-6

[39] Boin A , Couvelard A , Couderc C , et al. Proteomic screening identifies a YAP‐driven signaling network linked to tumor cell proliferation in human schwannomas. Neuro Oncol. 2014;16(9):1196‐1209. 24558021 10.1093/neuonc/nou020 PMC4136892

[40] Lin C , Yao E , Zhang K , et al. YAP is essential for mechanical force production and epithelial cell proliferation during lung branching morphogenesis. Elife. 2017;6:e21130. 28323616 10.7554/eLife.21130 PMC5360446

[41] Guerrant W , Kota S , Troutman S , et al. YAP mediates tumorigenesis in neurofibromatosis type 2 by promoting cell survival and proliferation through a COX‐2‐EGFR signaling axis. Cancer Res. 2016;76(12):3507‐3519. 27216189 10.1158/0008-5472.CAN-15-1144 PMC4911274

[42] Diamantopoulou Z , White G , Fadlullah MZH , et al. TIAM1 antagonizes TAZ/YAP both in the destruction complex in the cytoplasm and in the nucleus to inhibit invasion of intestinal epithelial cells. Cancer Cell. 2017;31(5):621‐634.e626. 28416184 10.1016/j.ccell.2017.03.007 PMC5425402

[43] Wang Y , Pan P , Wang Z , et al. beta‐catenin‐mediated YAP signaling promotes human glioma growth. J Exp Clin Cancer Res. 2017;36(1):136. 28962630 10.1186/s13046-017-0606-1 PMC5622484

[44] Mayrhofer M , Gourain V , Reischl M , et al. A novel brain tumour model in zebrafish reveals the role of YAP activation in MAPK‐ and PI3K‐induced malignant growth. Dis Model Mech. 2017;10(1):15‐28. 27935819 10.1242/dmm.026500 PMC5278524

[45] Zhang HT , Gui T , Sang Y , et al. The BET bromodomain inhibitor JQ1 suppresses chondrosarcoma cell growth via regulation of YAP/p21/c‐Myc signaling. J Cell Biochem. 2017;118(8):2182‐2192. 28059436 10.1002/jcb.25863

[46] Liu Z , Zeng W , Wang S , et al. A potential role for the Hippo pathway protein, YAP, in controlling proliferation, cell cycle progression, and autophagy in BCPAP and KI thyroid papillary carcinoma cells. Am J Transl Res. 2017;9(7):3212‐3223. 28804541 PMC5553873

[47] Jiang J , Chang W , Fu Y , et al. SAV1 represses the development of human colorectal cancer by regulating the Akt‐mTOR pathway in a YAP‐dependent manner. Cell Prolif. 2017;50(4):e12351. 10.1111/cpr.12351 PMC6529088 28618450

[48] Zhang Y , Yuan J , Zhang X , et al. Angiomotin promotes the malignant potential of colon cancer cells by activating the YAP‐ERK/PI3K‐AKT signaling pathway. Oncology Rep. 2016;36(6):3619‐3626. 10.3892/or.2016.5194 27779692

[49] Li XJ , Leem SH , Park MH , Kim SM . Regulation of YAP through an Akt‐dependent process by 3, 3'‐diindolylmethane in human colon cancer cells. Int J Oncol. 2013;43(6):1992‐1998. 24100865 10.3892/ijo.2013.2121

[50] Basu S , Totty NF , Irwin MS , Sudol M , Downward J . Akt phosphorylates the Yes‐associated protein, YAP, to induce interaction with 14‐3‐3 and attenuation of p73‐mediated apoptosis. Mol Cell. 2003;11(1):11‐23. 12535517 10.1016/s1097-2765(02)00776-1

[51] Garcia‐Gil A , Galan‐Enriquez CS , Perez‐Lopez A , Nava P , Alpuche‐Aranda C , Ortiz‐Navarrete V . SopB activates the Akt‐YAP pathway to promote *Salmonella* survival within B cells. Virulence. 2018;9(1):1390‐1402. 30103648 10.1080/21505594.2018.1509664 PMC6177241

[52] Mo JS , Park HW , Guan KL . The Hippo signaling pathway in stem cell biology and cancer. EMBO Rep. 2014;15(6):642‐656. 24825474 10.15252/embr.201438638 PMC4197875

[53] Schlegelmilch K , Mohseni M , Kirak O , et al. Yap1 acts downstream of alpha‐catenin to control epidermal proliferation. Cell. 2011;144(5):782‐795. 21376238 10.1016/j.cell.2011.02.031 PMC3237196

[54] von Gise A , Lin Z , Schlegelmilch K , et al. YAP1, the nuclear target of Hippo signaling, stimulates heart growth through cardiomyocyte proliferation but not hypertrophy. Proc Natl Acad Sci USA. 2012;109(7):2394‐2399. 22308401 10.1073/pnas.1116136109 PMC3289361

[55] Tian W , Yu J , Tomchick DR , Pan D , Luo X . Structural and functional analysis of the YAP‐binding domain of human TEAD2. Proc Natl Acad Sci USA. 2010;107(16):7293‐7298. 20368466 10.1073/pnas.1000293107 PMC2867681

[56] Zhou Z , Hu T , Xu Z , et al. Targeting Hippo pathway by specific interruption of YAP‐TEAD interaction using cyclic YAP‐like peptides. FASEB J. 2015;29(2):724‐732. 25384421 10.1096/fj.14-262980

[57] Marti P , Stein C , Blumer T , et al. YAP promotes proliferation, chemoresistance, and angiogenesis in human cholangiocarcinoma through TEAD transcription factors. Hepatology. 2015;62(5):1497‐1510. 26173433 10.1002/hep.27992

[58] Brodowska K , Al‐Moujahed A , Marmalidou A , et al. The clinically used photosensitizer Verteporfin (VP) inhibits YAP‐TEAD and human retinoblastoma cell growth in vitro without light activation. Exp Eye Res. 2014;124:67‐73. 24837142 10.1016/j.exer.2014.04.011 PMC4135181

[59] Zhang W , Gao Y , Li P , et al. VGLL4 functions as a new tumor suppressor in lung cancer by negatively regulating the YAP‐TEAD transcriptional complex. Cell Res. 2014;24(3):331‐343. 24458094 10.1038/cr.2014.10 PMC3945886

[60] Smith SA , Sessions RB , Shoemark DK , et al. Antiproliferative and antimigratory effects of a novel YAP‐TEAD interaction inhibitor identified using in silico molecular docking. J Med Chem. 2019;62(3):1291‐1305. 30640473 10.1021/acs.jmedchem.8b01402 PMC6701825

[61] Chu IM , Hengst L , Slingerland JM . The Cdk inhibitor p27 in human cancer: prognostic potential and relevance to anticancer therapy. Nat Rev Cancer. 2008;8(4):253‐267. 18354415 10.1038/nrc2347

[62] Morishita D , Katayama R , Sekimizu K , Tsuruo T , Fujita N . Pim kinases promote cell cycle progression by phosphorylating and down‐regulating p27Kip1 at the transcriptional and posttranscriptional levels. Cancer Res. 2008;68(13):5076‐5085. 18593906 10.1158/0008-5472.CAN-08-0634

[63] Sekimoto T , Fukumoto M , Yoneda Y . 14‐3‐3 suppresses the nuclear localization of threonine 157‐phosphorylated p27(Kip1). EMBO J. 2004;23(9):1934‐1942. 15057270 10.1038/sj.emboj.7600198 PMC404318

[64] Chu I , Sun J , Arnaout A , et al. p27 phosphorylation by Src regulates inhibition of cyclin E‐Cdk2. Cell. 2007;128(2):281‐294. 17254967 10.1016/j.cell.2006.11.049 PMC1961623

[65] Hattori T , Isobe T , Abe K , et al. Pirh2 promotes ubiquitin‐dependent degradation of the cyclin‐dependent kinase inhibitor p27Kip1. Cancer Res. 2007;67(22):10789‐10795. 18006823 10.1158/0008-5472.CAN-07-2033

[66] Zhao P , Aguilar AE , Lee JY , et al. Sphingadienes show therapeutic efficacy in neuroblastoma in vitro and in vivo by targeting the AKT signaling pathway. Invest New Drugs. 2018;36(5):743‐754. 29335887 10.1007/s10637-017-0558-5 PMC6047934

