# Supplementary figures and images for: YAP promotes the proliferation of neuroblastoma cells through decreasing the nuclear location of p27Kip1 mediated by Akt
Source: Cell Prolif. 2019 Dec 20;53(2):e12734. doi: 10.1111/cpr.12734 (PMC7046475; doi:10.1111/cpr.12734)

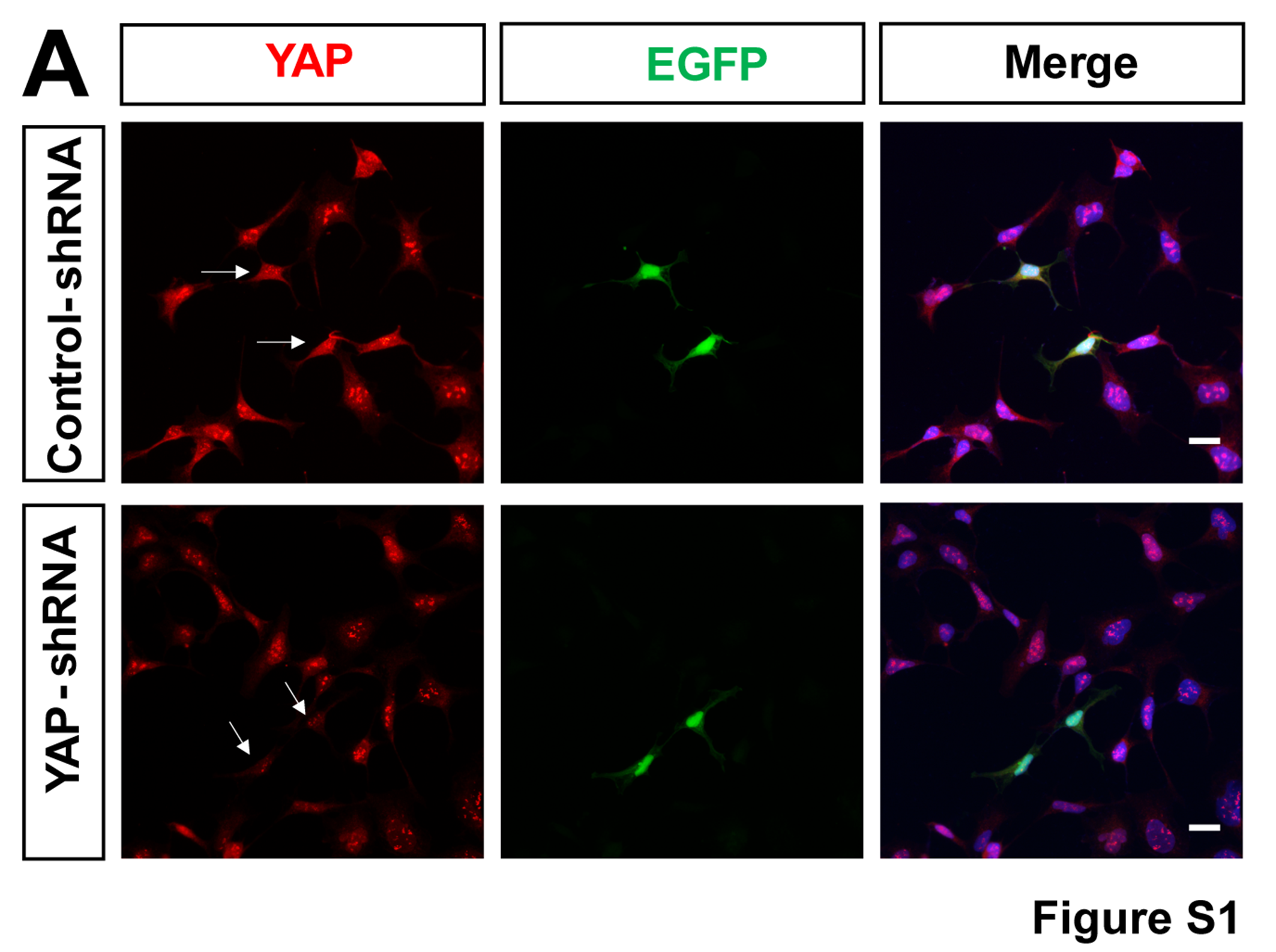

Supplement: Supplementary file 1 [file CPR-53-e12734-s001.tif]

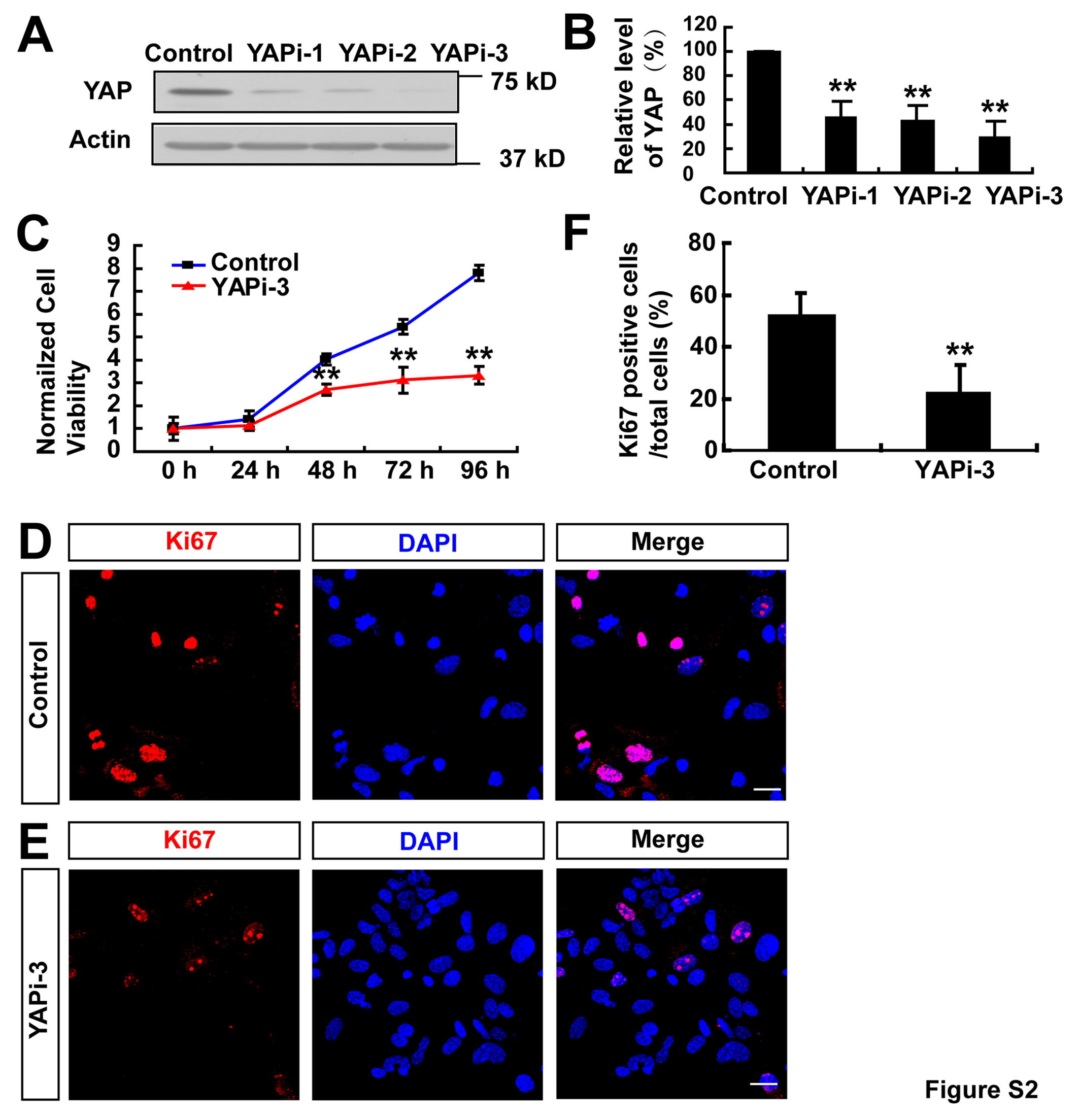

Supplement: Supplementary file 2 [file CPR-53-e12734-s002.tif]

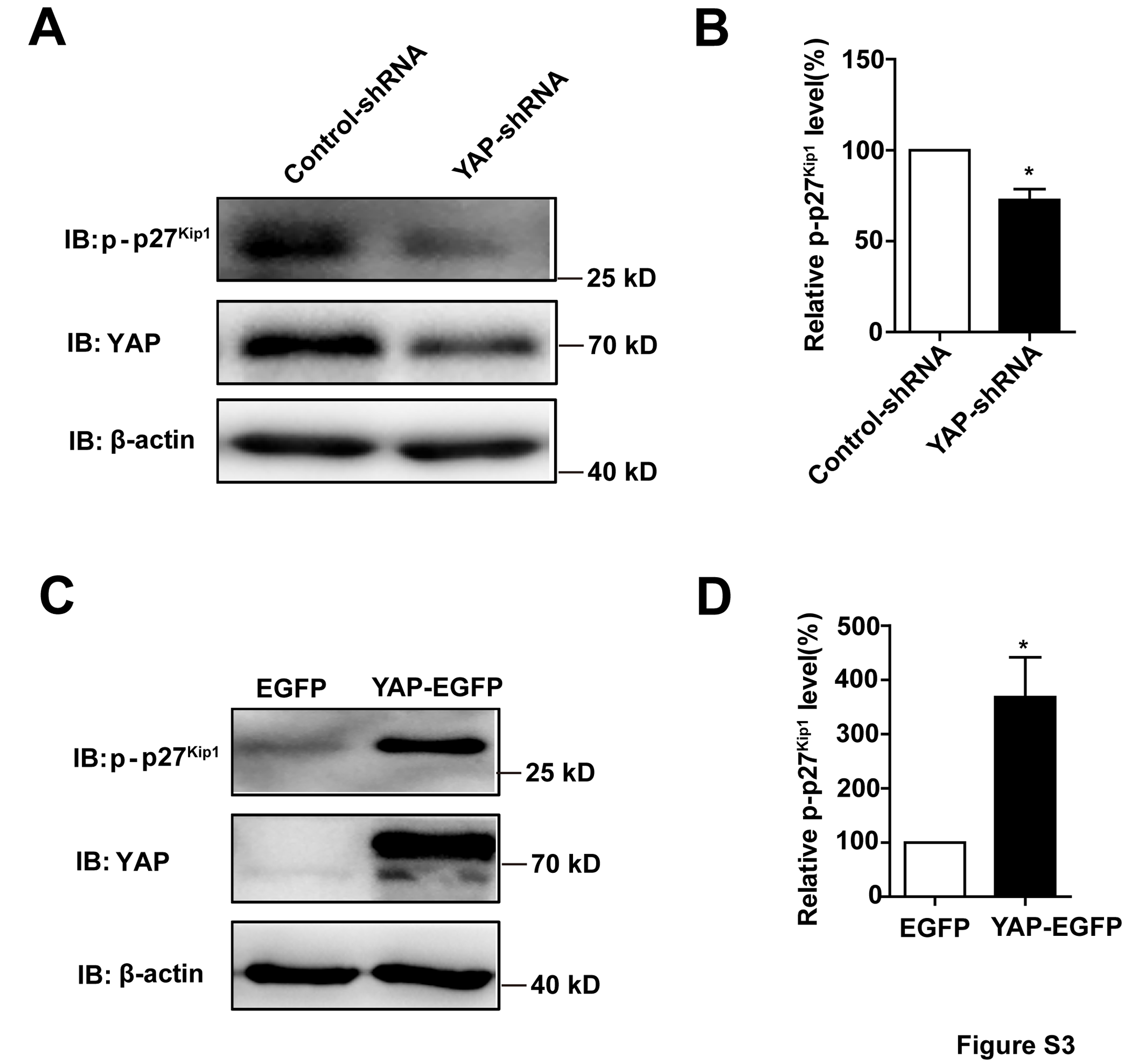

Supplement: Supplementary file 3 [file CPR-53-e12734-s003.tif]

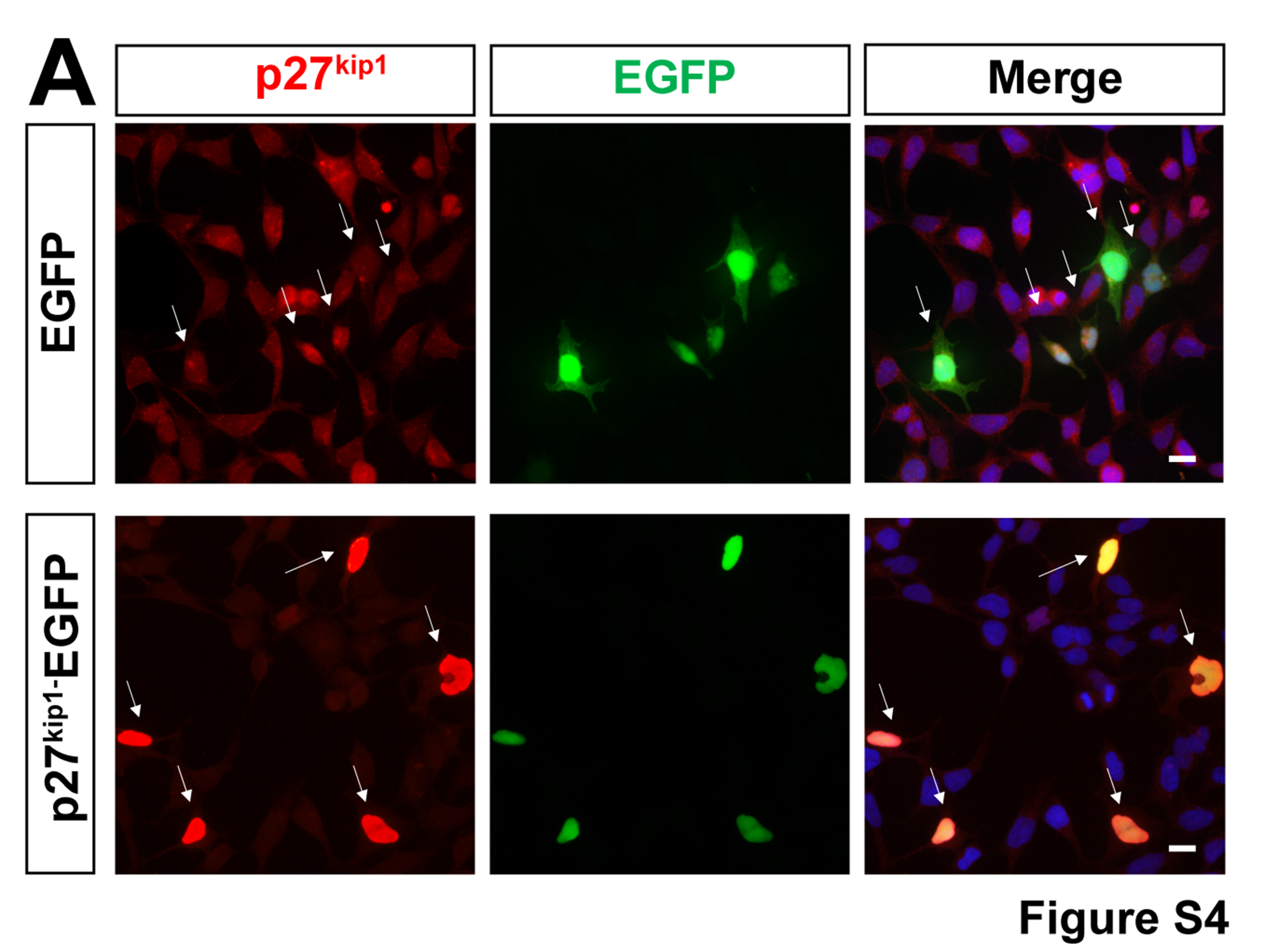

Supplement: Supplementary file 4 [file CPR-53-e12734-s004.tif]

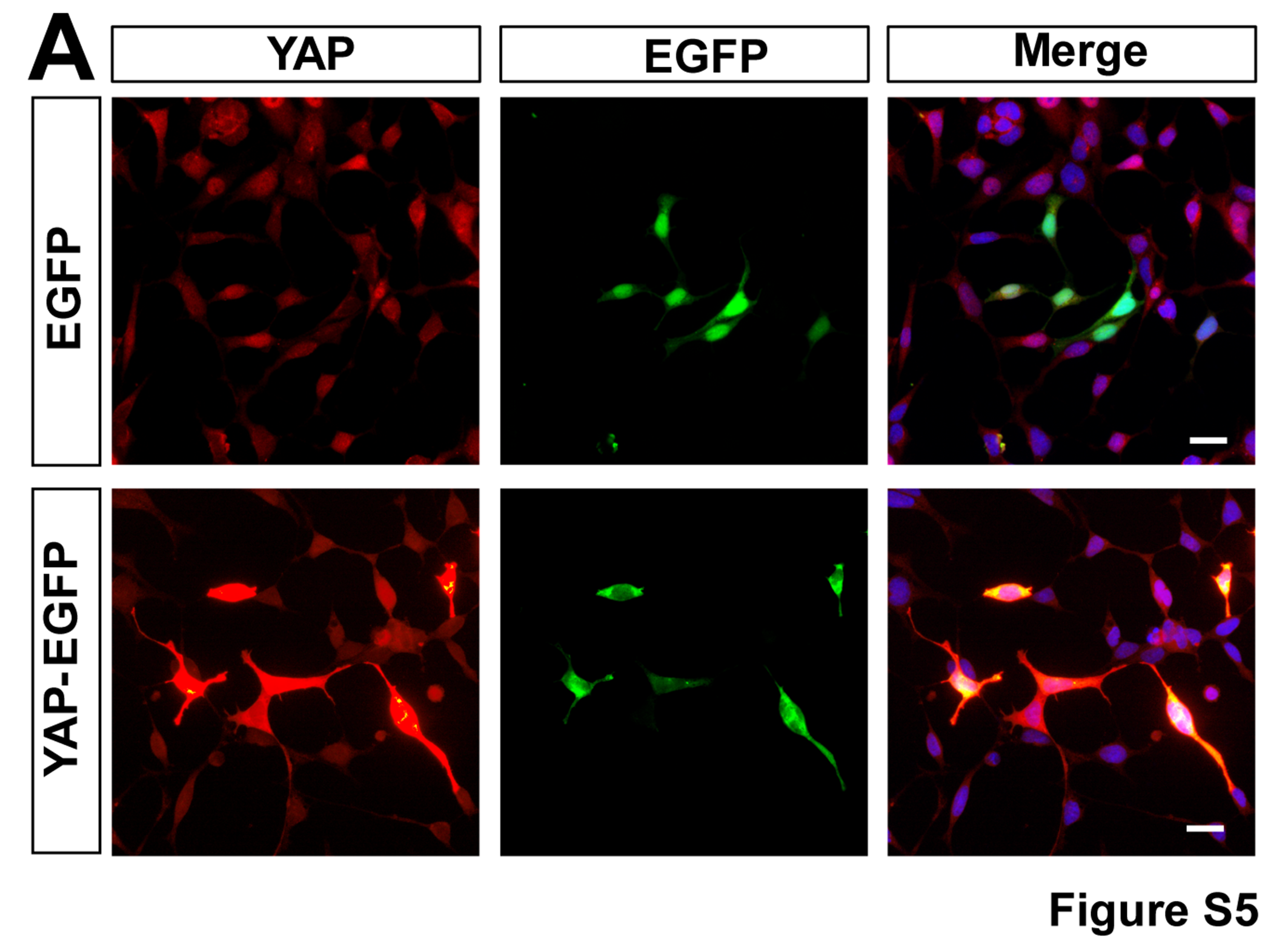

Supplement: Supplementary file 5 [file CPR-53-e12734-s005.tif]

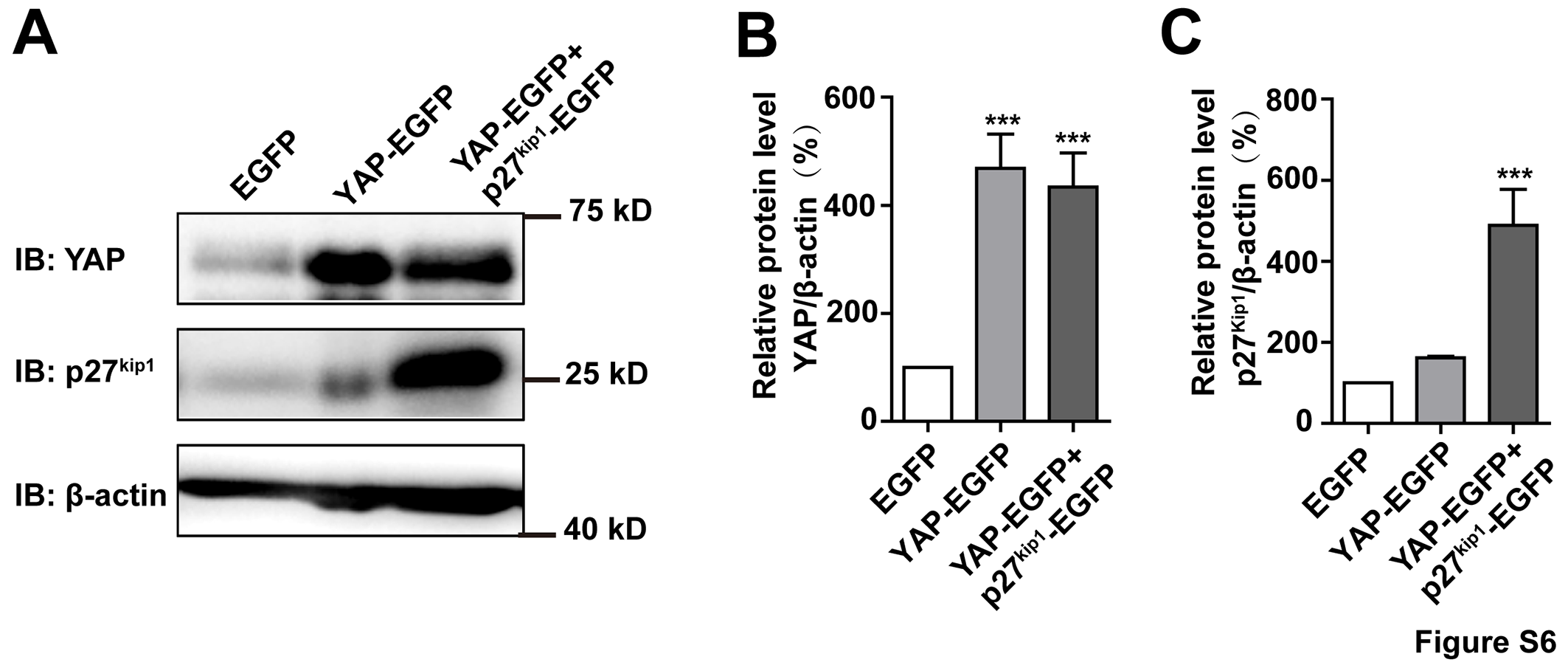

Supplement: Supplementary file 6 [file CPR-53-e12734-s006.tif]

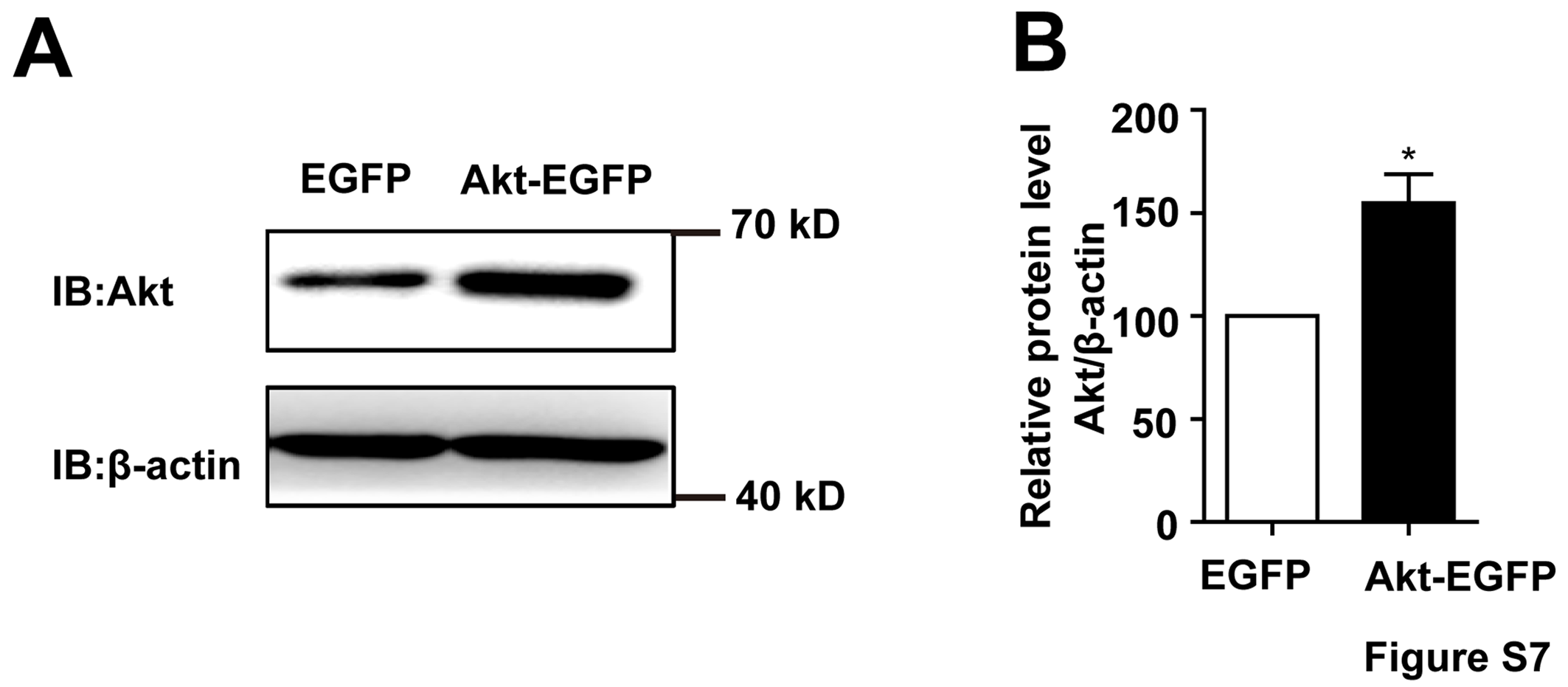

Supplement: Supplementary file 7 [file CPR-53-e12734-s007.tif]

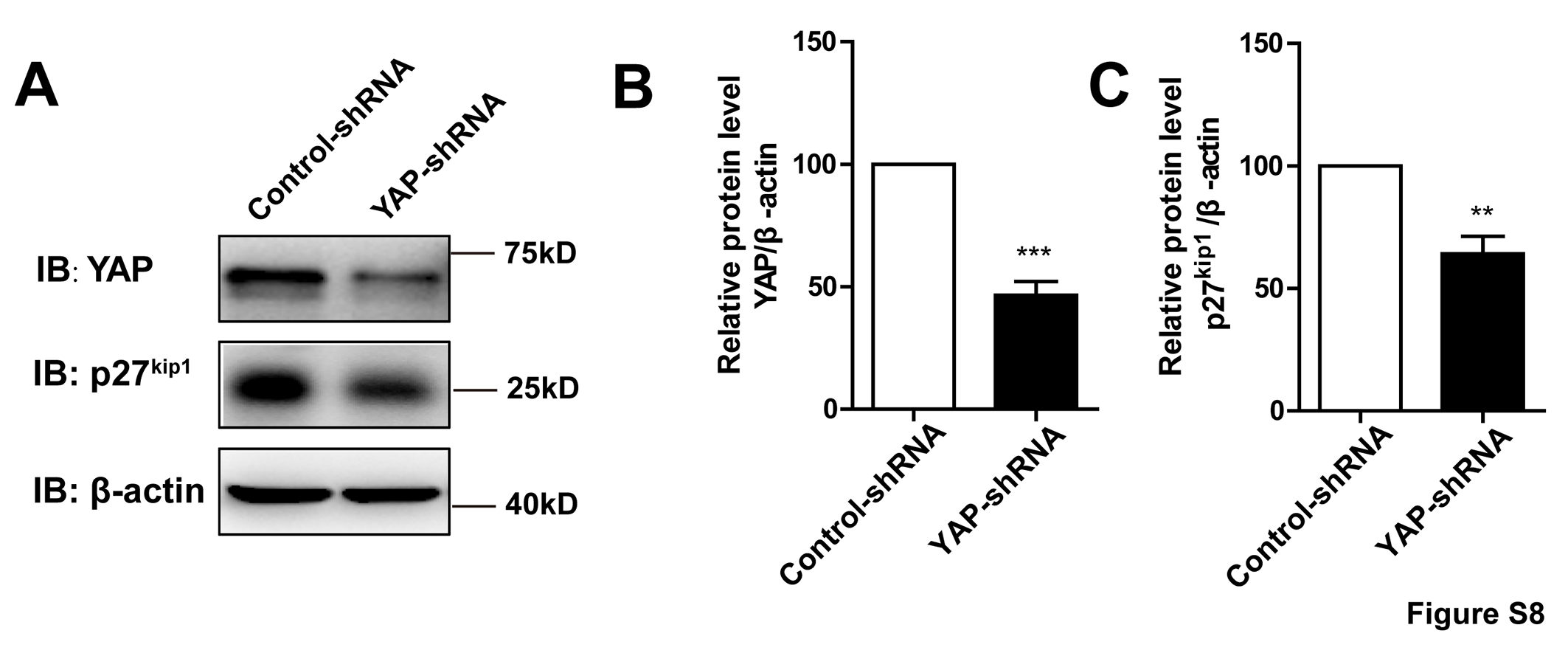

Supplement: Supplementary file 8 [file CPR-53-e12734-s008.tif]

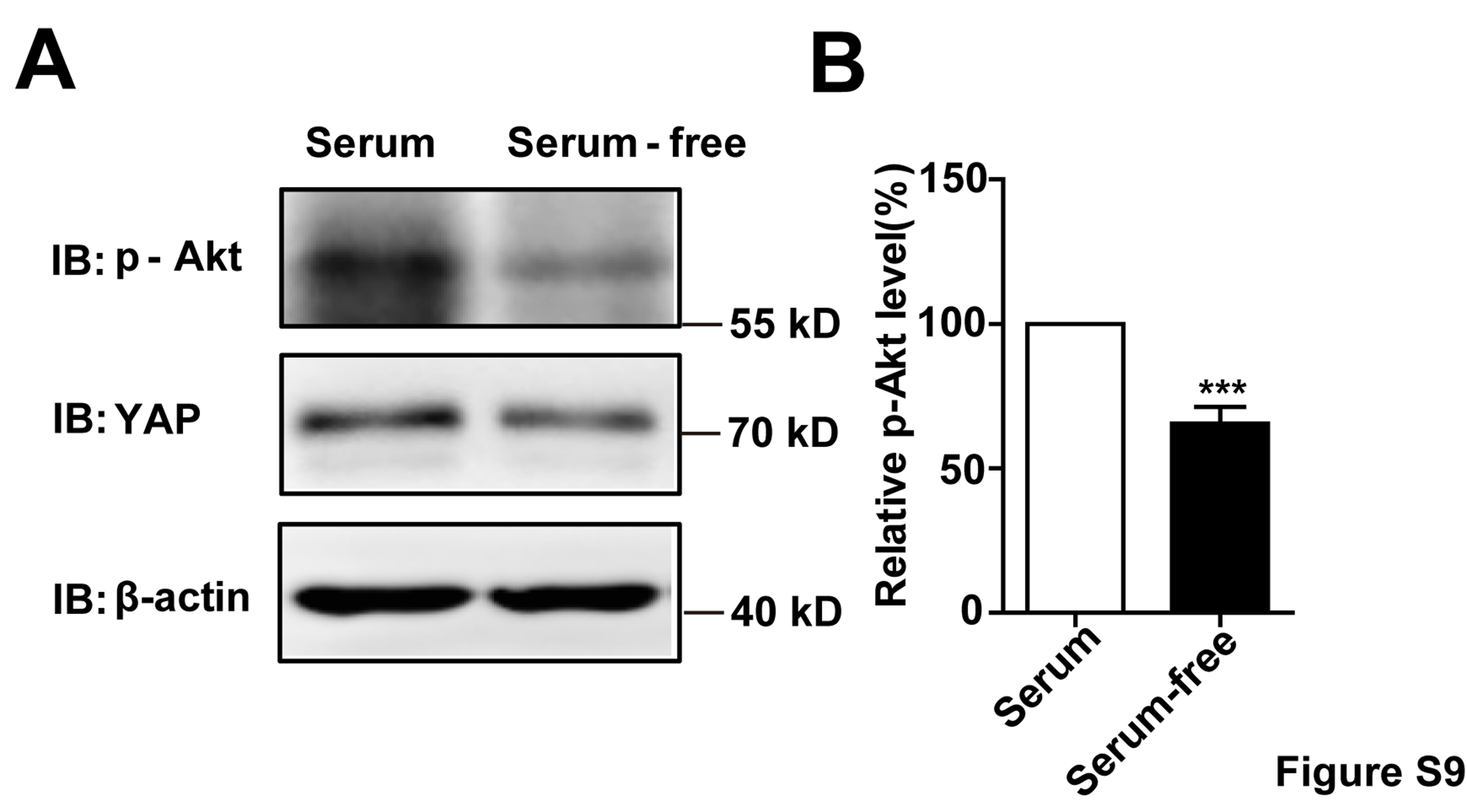

Supplement: Supplementary file 9 [file CPR-53-e12734-s009.tif]
