# Supplementary material for: YAP promotes the proliferation of neuroblastoma cells through decreasing the nuclear location of p27Kip1 mediated by Akt
Source: Cell Prolif. 2019 Dec 20;53(2):e12734. doi: 10.1111/cpr.12734 (PMC7046475; doi:10.1111/cpr.12734)
Supplement: Supplementary file 10 [file CPR-53-e12734-s010.docx]

**SUPPLEMENTAL DATA**

**FIGURE S1** Identification of the efficiency of YAP-shRNA constructs in SH-SY5Y cell lines by immunostaining. A, Immunostaining of YAP (red) showed knockdown efficiency of YAP. SH-SY5Y cells were co-transfected with vectors encoding control-shRNA, EGFP or YAP-shRNA, EGFP for 48-72 h. Arrows indicated control cells or YAP down-regulated cells. Scale bars, 20 μm.

**FIGURE S2 YAP knockdown inhibited the proliferation of SH-SY5Y cells.**

A, Representative western blot image of the expression of YAP in SH-SY5Y cells transfected with three YAP-shRNA sequences (YAPi-1, YAPi-2, YAPi-3) for 48-72 h. B, Quantification of YAP expression as shown in A (n=3). C, The effects of YAP knockdown by YAPi-3 on SH-SY5Y cell viability detected by CCK-8 (n=3). D, E, Immunostaining analysis of Ki67 (red) in SH-SY5Y cells transfected with control-shRNA, or YAPi-3 constructs for 48 h. F, Quantitative analysis of the percentages of Ki67^+^ positive cells over total cells as shown in D, E (n=12). Scale bars, 20 μm. Data were mean ± s.e.m. *^**^P < 0.01*.

**FIGURE** **S3** YAP regulated the p-p27^kip1^ level in SH-SY5Y cells. A, Representative western blot image of the expression of p-p27^kip1^ and YAP in SH-SY5Y cells transfected with EGFP or YAP-EGFP for 48 h. B, Quantification of p-p27^kip1^ expression as shown in A (n=6). C, Representative western blot image of the expression of p-p27^kip1^ and YAP in SH-SY5Y cells transfected with vectors encoding control-shRNA or YAP-shRNA for 48-72 h. D, Quantification of p-p27^kip1^ expression as shown in C (n=4). Data were mean ± s.e.m. *^*^P < 0.05*.

**FIGURE S4** Identification of p27^kip1^-EGFP and EGFP constructs in SH-SY5Y cell lines by immunostaining. A, Immunostaining of p27kip1 (red) showed the overexpression efficiency of p27^kip1^. SH-SY5Y cells were transfected with vectors encoding EGFP or p27^kip1^-EGFP for 48-72 h. Arrows indicated that the transfected p27^kip1^ were mainly located in the nucleus. Scale bars, 20 μm.

**FIGURE S5** Identification of YAP-EGFP and EGFP constructs in SH-SY5Y cell lines by immunostaining. A, Immunostaining of YAP (red) showed the overexpression efficiency of YAP. SHSY5Y cells were transfected with vectors encoding EGFP or YAP-EGFP for 48-72 h. Scale bars, 20 μm.

**FIGURE** **S6** Identification of p27^kip1^-EGFP, YAP-EGFP, and EGFP constructs in SH-SY5Y cell lines by western blot. A, Representative western blot image of the expression of YAP and p27^kip1^ in SH-SY5Y cells transfected with vectors encoding EGFP, YAP-EGFP, or YAP-EGFP plus p27^kip1^-EGFP for 48-72 h. B and C, Quantification of YAP and p27^kip1^ expression as shown in A (n=3). Data were mean ± s.e.m. ****P < 0.001*.

**FIGURE** **S7** Identification of Akt-EGFP and EGFP constructs in SH-SY5Y cell lines by western blot. A, Representative western blot image of the expression of Akt in SH-SY5Y cells transfected with vectors encoding EGFP or Akt-EGFP for 48-72 h. B, Quantification of Akt expression as shown in A (n=3). Data were mean ± s.e.m. **P< 0.05.*

**FIGURE** **S8** Knockdown of YAP reduced the total level of p27^kip1^ in SH-SY5Y cells. A, Representative western blot image of the expression of YAP and p27^kip1^ in SH-SY5Y cells transfected with vectors encoding control-shRNA or YAP-shRNA for 48-72 h. B, C, Quantification of YAP and p27^kip1^ expression as shown in A (n=15). Data were mean ± s.e.m. *^**^P < 0.01*, *^***^P < 0.001*.

**FIGURE S9** Serum starvation decreased p-Akt level significantly in SH-SY5Y cells. A, Representative western blot image of the expression of p-Akt and YAP in SH-SY5Y cells treated with serum-containing or serum-free medium for 24 h. B, Quantification of p-Akt expression as shown in A (n=4). Data were mean ± s.e.m. *^***^P < 0.001*.
